# NOD-like receptors in asthma

**DOI:** 10.3389/fimmu.2022.928886

**Published:** 2022-09-14

**Authors:** Daniel Alvarez-Simon, Saliha Ait Yahia, Patricia de Nadai, Camille Audousset, Mathias Chamaillard, Ivo Gomperts Boneca, Anne Tsicopoulos

**Affiliations:** ^1^ Univ. Lille, CNRS, Inserm, CHU Lille, Institut Pasteur de Lille, U1019-UMR9017-CIIL-Centre d’Infection et d’Immunité de Lille, Lille, France; ^2^ Laboratory of Cell Physiology, INSERM U1003, University of Lille, Lille, France; ^3^ Institut Pasteur, Université Paris Cité, CNRS UMR 6047, INSERM U1306, Unité Biologie et génétique de la paroi bactérienne, Paris, France

**Keywords:** asthma, innate immunity, NOD1, NOD2, RIPK2, NLRP3, therapy

## Abstract

Asthma is an extremely prevalent chronic inflammatory disease of the airway where innate and adaptive immune systems participate collectively with epithelial and other structural cells to cause airway hyperresponsiveness, mucus overproduction, airway narrowing, and remodeling. The nucleotide-binding oligomerization domain (NOD)-like receptors (NLRs) are a family of intracellular innate immune sensors that detect microbe-associated molecular patterns and damage-associated molecular patterns, well-recognized for their central roles in the maintenance of tissue homeostasis and host defense against bacteria, viruses and fungi. In recent times, NLRs have been increasingly acknowledged as much more than innate sensors and have emerged also as relevant players in diseases classically defined by their adaptive immune responses such as asthma. In this review article, we discuss the current knowledge and recent developments about NLR expression, activation and function in relation to asthma and examine the potential interventions in NLR signaling as asthma immunomodulatory therapies.

## Introduction

Asthma is the most prevalent chronic airway disorder and as such represents a major health and socioeconomic burden (1). Asthma is characterized by recurring symptoms of reversible airflow obstruction, airway hyperresponsiveness (AHR), and inflammatory processes leading to the fluctuating recruitment of eosinophils and/or neutrophils. Asthma clinical manifestations can vary from mild to severe, and the phenotypical presentation is very heterogeneous. This diversity in clinic presentation reflects the complexity of the different basic mechanisms that lead to asthma development. Despite being classically considered the hallmark Th2 disease of the lung, the underlying mechanisms of asthma are now known to be much more complex than previously thought. Even in the cases where is present an obvious dominant type 2 immune adaptive response, this is not only orchestrated by adaptive T cells, but also by different types of innate and structural cells (2) and different antigen-independent innate immune mechanisms that play critical roles in the disease progression and outcome (3). Following this conception of asthma being driven by Th2 responses, most part of the asthma research over the years has focused on adaptive immune and antigen-dependent responses. However, in recent times innate immune mechanisms have increasingly been found to play pivotal roles in asthma pathogenesis, by shaping downstream adaptive responses (4, 5).

The epithelium of the airway represents the largest human mucosal surface exposed to the outside environment, and therefore, it is continuously in contact with different external and internal stimuli. In this context, the innate immune system represents the first line of response and defense against the different environmental signals, including pathogens, allergens, particles, and chemicals. In order to maintain the delicate balance between the clearance of the harmful stimuli and avoiding chronic inflammation, the innate immune system is equipped with extracellular and intracellular sensors called pattern recognition receptors (PRR). PRR are a class of receptors that can directly recognize specific microbial molecular signatures including microbe-associated molecular patterns (MAMP), endogenous damage-associated molecular patterns (DAMP), as well as stress signals (6). The recognition and binding of their ligands by PRR leads to the recruitment of respective adaptor molecules and results in the initiation of downstream signaling pathways that represent a bridge between nonspecific immunity and specific immunity (7). Therefore, the increasing understanding of these different signaling pathways and the implication of different PRR receptor families, such as the (NOD)-like receptors (NLR) in the mechanisms leading to asthma development and other immune-mediated diseases, have resulted in their acknowledgment as much more than innate sensors but relevant players in asthma.

In this review article, we will discuss the current knowledge and recent developments about NLR expression, activation, and function concerning asthma, and examine the potential therapeutic interventions in NLR signaling as asthma immunomodulatory drugs.

## The NLR receptor family

**Figure 1:**
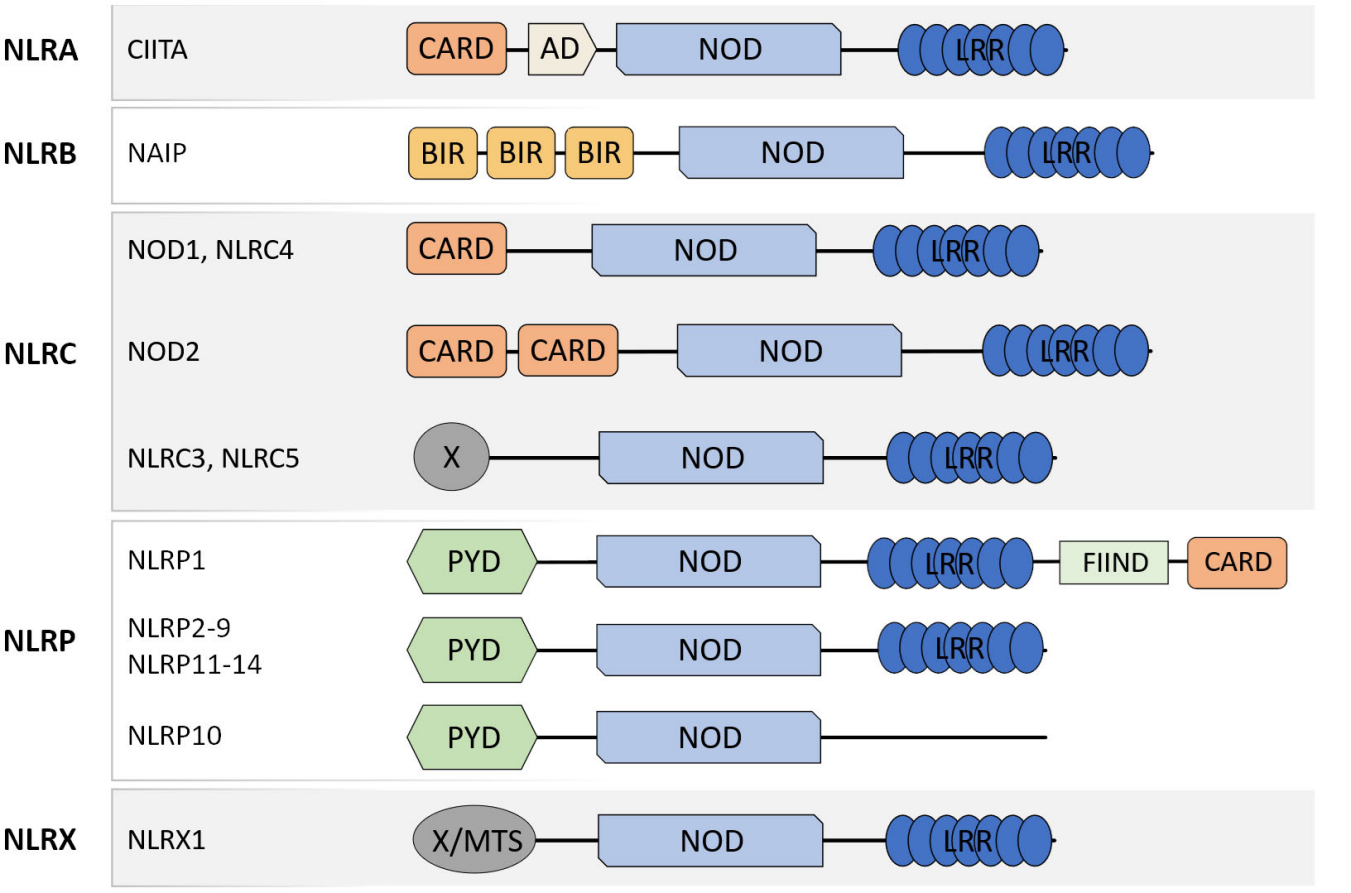
Schematic representation of molecular domain structures of the different NLR subfamilies. NLR present a common domain structure with an N-terminal effector domain, a central NOD domain, and a C-terminal LRR domain. The different effector domains allow the categorization of NLR into five subfamilies: NLRA, NLRB, NLRC, NLRP, and NLRX. CARD caspase recruitment domain, AD acidic transactivation domain, NOD nucleotide-binding oligomerization domain, LRR leucine-rich repeat, PYD pyrin domain, FIIND function to find domain, X unidentified.

NLR are a family of PRR well-recognized for their central roles in the maintenance of tissue homeostasis and host defense against bacteria, viruses and fungi through the detection of MAMP and DAMP. The members of the NLR family present similar molecular architecture with a C-terminal leucine-rich repeat (LRR) domain, involved in ligand recognition, a central nucleotide-binding oligomerization domain (NOD) that facilitates self-oligomerization and adenosine triphosphate (ATP)-dependent NLR activation, and a variable N-terminal effector domain that will bind to the adaptor molecules and downstream effectors to mediate signal transduction. There are four types of effector domains with unique functional characteristics that allow the categorization of NLR into five different subfamilies: NLRA, NLRB, NLRC, NLRP, and NLRX (**Figure 1**).

### NLRA

The Major histocompatibility complex (MHC) class II transactivator (CIITA) is the only known member of the NLRA subfamily and the founding member of the NLR family (8). CIITA has been recognized as the “master regulator” of MHCII expression and as such is responsible for the regulation of the expression of MHCII in different cell populations including professional antigen-presenting cells (9).

#### Structure

CIITA protein’s structure is characterized by the presence of an N-terminal acidic transactivation domain (AD) and a region rich in prolines, serines, and threonines (P/S/T). At least three different CIITA isoforms (I, III and IV) differing in their N terminal domains are expressed under the control of different promoters (10). However, while all the CIITA isoforms share the aforementioned AD and P/S/T domains, only isoform I presents an N terminal caspase activation and recruitment domain (CARD), that could participate in enhancing MHCII transcription (10, 11).

#### Signaling

CIITA expression has been reported to be necessary and sufficient to induce MHCII gene expression *via* self-association, oligomerization, and nuclear translocation (12). Despite being sufficient for MHCII expression, CIITA direct binding to DNA has never been confirmed yet and for that reason it has frequently been described as a co-activator, inducing transcription initiation and elongation through multiple mechanisms such as the recruitment of components of the general transcription machinery, RNA polymerase II phosphorylation, and the recruitment of other different chromatin remodeling co-activators (11).

#### Expression

CIITA is mainly regulated at the transcriptional level (13). As expected due to its role in the control of MHCII expression, CIITA is constitutively expressed in professional antigen-presenting cells such as dendritic cells, macrophages, B cells and also thymic epithelial cells (11, 14, 15). Besides this constitutive expression in antigen-presenting cells, CIITA expression can be induced by different stimuli, notably IFNγ (15). Despite being expressed in other epithelial surfaces such as the gut epithelium, CIITA expression is not detectable in the human bronchial epithelial cells of healthy individuals. However, it has been shown that in the bronchial epithelium of asthmatic patients or in the case of viral infections MHCII expression is enhanced in a CIITA-dependent manner (16).

### NLRB

As is the case with the NLRA subfamily, in humans the NLRB subfamily consists of only one known member the Neuronal apoptosis inhibitory protein (NAIP).

#### Structure

NAIP protein´s structure is characterized by the presence of N-terminal baculoviral inhibition of apoptosis (BIR) domains, followed by the NOD domain and several LRRs.

#### Signaling

Upon interaction with an activating bacteria-derived ligand, such as flagellin or T3SS needle, NAIP is subjected to a conformational change in an active form able to interact with NLRC4, another NLR family member. The interaction of ligand bound NAIP with an NLRC4 monomer triggers conformational changes in NLRC4, driving it to an active form (17). This activation of the first NLRC4 monomer allows the recruitment of additional NLRC4 monomers and leads to a self-propagation mechanism resulting in the formation of an inflammasome able to recruit and activate caspase-1, that will process the proforms of interleukin(IL)1β and IL18 cytokines (17, 18).

#### Expression

NAIP expression has been detected in human monocytes and macrophages especially in the context of bacterial infections (19, 20). Increasing evidence suggests that other myeloid cells can express NAIP, such as dendritic cells (21) and neutrophils (19). The detection of NLRC4 expression and NLRC4 inflammasome responses in astrocytes and microglia, suggests the expression of NAIP in the human brain (22). Furthermore, NAIP expression has also been detected in the intestinal epithelial cells (23, 24). In the lung, the expression of NAIP in alveolar macrophages has been confirmed by several authors in the context of bacterial infections (25–27). However, despite the existence of reports supporting NAIP expression in bacterial infected alveolar epithelium cell lines (27), no solid evidence supports the expression of NAIP in the human bronchial lung epithelium (26).

### NLRC

The human NLRC is the second most numerous of the NLR subfamilies, consisting of five members (NLRC1-5). NLRC1 and NLRC2, best known as NOD1 and NOD2 respectively, are considered the two primary members of the NLRC subfamily (28, 29).

#### Structure

The members of the NLRC subfamily are characterized by the presence of a CARD domain. Despite the apparent absence of a CARD domain (see **Figure 1**), NLRC3 and NLRC5 are considered part of the NLRC subfamily due to their homology and phylogenetic relationship with the other members. Furthermore, although the N-terminus has unusual structural features, solution Nuclear Magnetic Resonance analysis confirmed its relationship to CARD domains (atypical CARD) (30, 31).

#### Signaling

**Figure 2:**
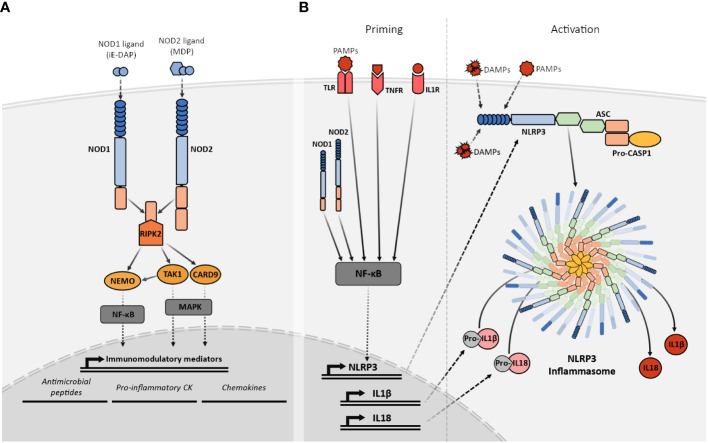
Simplified representation of the NOD1, NOD2, and NLRP3 signaling pathways. **(A)** Recognition of their specific PGN fragments agonists through their LRR domains activates the NOD1 and NOD2 receptors. Their activation facilitates the recruitment of RIPK2 that subsequently interacts with TAK1 or NEMO triggering the NF-κB or MAPK pathways. The NF-κB and MAPK pathways, stimulate the transcriptional upregulation of different types of immunomodulatory mediators such as antimicrobial peptides, proinflammatory cytokines and chemokines. **(B)** NLRP3 activation requires two signals. The priming (left) is provided by MAMPs, DAMPs, or pro-inflammatory cytokines leading to the transcriptional upregulation of NLRP3, other inflammasome components, pro-IL1β and pro-IL18 through NF-κB signaling. The activation signal (right) is provided by any of the numerous MAMPs or DAMPs capable of NLRP3 activation such as ATP, particulates, and crystals causing the NLRP3 inflammasome complex assembly and subsequent cleavage of pro-IL1β and pro-IL18 into their mature forms by active caspase-1.

After ligand recognition, the NLRC members activate different signaling pathways *via* CARD-CARD interactions with different adaptor proteins. Although NOD1 and NOD2 recognize different ligands through their LRR domains, both act downstream *via* the same adaptor protein the Receptor-interacting serine/threonine-protein kinase 2 (RIPK2) (29, 32). Their activation and signaling through RIPK2 participate in the regulation of very relevant pathways involved in a variety of cellular responses, including inflammatory responses *via* activation of NF-κB, and MAPKs (33, 34). NOD1 and NOD2 exist in an inactive conformation where the LRR is folded over the NOD and CARD domains preventing dimerization and the CARD engagement with RIPK2. Upon ligand recognition by the LRR, the NOD1 and NOD2 adopt “open conformations” that allow the oligomerization and subsequent signaling (35). The interaction of NOD1 and NOD2 with RIPK2 *via* CARD domains results in the activation of the two aforementioned signaling pathways by binding RIPK2 to the NF-κB essential modulator kinase (NEMO) with subsequent NF-κB activation (36), and TAK1 or CARD9 leading to MAPK signaling (33, 37) (**Figure 2A**). Furthermore, the NOD1 or NOD2-dependent activation of both of these signaling pathways can interact with other PRR such as the Toll-like receptors (TLR) (38).

There is also evidence indicating that NOD1 and NOD2 could be able to activate signaling response in a RIPK2-independent manner. NOD1 and NOD2 are able to induce type I interferon signaling in response to the aforementioned detection of viral RNA, suggesting a role for NOD1 and NOD2 in antiviral immunity (39–41). The activation of NOD1 and NOD2 has been also related to the initiation of autophagy in a RIPK2-independent manner. It has been proposed that the triggering mediator in autophagy could be ATG16L1 and that this process could be related to intracellular bacteria and viral clearance (42). NOD1 and NOD2 activation through all these signaling pathways result in an array of immunomodulatory effects that are important for host defense, tissue homeostasis, and the shaping of adaptive responses through the production of antimicrobial peptides, proinflammatory cytokines, and chemokines among other mechanisms (43, 44).

The differences in the LRR domains of NOD1 and NOD2 are responsible for their ligand specificity (45, 46) and this results in differential ligand recognition. NOD1 and NOD2 recognize different fragments derived from the degradation of peptidoglycan (PGN), the major component of the bacterial cell wall. PGN is a heteropolymer composed of a glycan backbone of a repeating GlcNAc-ß 1,4-MurNAc disaccharide. The MurNAc residues are modified with stem peptides that crosslink distinct glycans. The PGN fragments, also called muropeptides, capable of activating NOD1, contain a minimal γ-D-glutamyl-meso-diaminopimelic acid (iE-DAP) dipeptide core, which is predominantly found in Gram-negative, but also in some Gram-positive bacteria such as *Listeria monocytogenes* and *Bacillus* spp (47, 48). Unlike NOD1, NOD2 strictly requires the MurNAc residue to recognize PGN fragments. The most well-known NOD2 ligand is the muramyl dipeptide (MDP) which is broadly produced in both Gram-positive and Gram-negative bacteria (49). In contrast to what happens in the case of NOD1, the length of the peptide fragment is not critical for NOD2 recognition, and therefore NOD2 can be activated by a broader range of muropeptides from both Gram-negative and Gram-positive bacteria (49). Some authors had also provided evidence suggesting that NOD1 and NOD2 can also be indirectly activated in a NOD domain-dependent manner by molecules other than the PGN fragments, such as viral RNA (39, 41). Recently, it has been shown that sphingosine-1P is also an endogenous ligand by binding to the NOD domain (50). Furthermore, new evidence has suggested that NOD1 and NOD2, can also respond to danger signals such as disturbances in endoplasmic reticulum (ER) function leading to ER stress with an accumulation of misfolded proteins (51), however, a direct implication for ER stress in NOD1 and NOD2 activation is still a matter of controversy within the field (50, 52).

NLRC3 negatively regulates PI3K/mT0R pathway in epithelial cells (53), and NF-κB signaling (54) and the STING pathway (55, 56) in macrophages and T cells. Furthermore, NLRC3 is implicated in the activation, proliferation, and cytokine production of CD4^+^ T cells, participating directly in T cell regulation (57) as well as indirectly *via* its expression in dendritic cells (58).

NLRC4 is another relevant member of the NLRC subfamily. Despite similarities in the molecular structure, contrary to NOD1 and NOD2, NLRC4 induces the formation of an inflammasome complex. NLRC4 is mainly regulated at the transcriptional level and its expression is activated by pro-inflammatory stimuli such as TNFα (59). As mentioned before, ligand recognition by NAIP will result in an interaction with NLRC4 and subsequent inflammasome activation with recruitment and activation of caspase-1 and cleavage of pro-IL1β and pro-IL18 (19).

NLRC5 has been described to be a regulator of MHC class I genes. It has been proposed that similarly to CIITA, NLRC5 expression could be induced by IFNγ and regulated at the transcriptional level (60). NLRC5 protein translocates to the nucleus where it interacts with a specific MHC class I enhanceosome. It exists reported evidence suggesting a potential role for NLRC5 in innate immune regulation, such as NF-κB signaling regulation or type I interferon signaling (61). However, further investigation will be needed to confirm the NLRC5 implication beyond MHCI regulation.

#### Expression

NOD1 shows a fairly ubiquitous pattern of expression including lung epithelial cells, endothelial cells, airway smooth muscle cells, and different types of leukocytes (28, 62–65). In contrast, NOD2 expression is more restricted being highly expressed in myeloid cells, including macrophages (29) and dendritic cells (66), but also in human bronchial epithelial cells (67, 68).

NLRC3 is widely expressed and has been detected in both epithelial and immune cells with myeloid and lymphoid origin (54), but the highest levels of expression in humans have been detected in T cells and secondary lymphoid organs (57).

Together with NAIP, NLRC4 expression has been documented in human monocytes, macrophages, and dendritic cells (19–21). Moreover, NLRC4 is expressed in the brain (22) and in the intestinal epithelial cells (23, 24).

NLRC5 is expressed in a variety of different cells and tissues. High levels of expression of NLRC5 have been detected in spleen, lymph nodes, bone marrow, thymus, lung, and intestine (69, 70).

### NLRP

The NLRP is the largest of the NLR subfamilies with 14 members. At least five of the NLRP members, NLRP1, NLRP3, NLRP6, NLRP7, and NLRP12 are able to induce the formation of inflammasome complexes and regulate the cleavage and release of IL1β and IL18 in response to different MAMP or DAMP (71). Among them, the most well-studied are NLRP1 and NLRP3.

#### Structure

The prototypic NLRP protein structure is characterized by the presence of an N-terminal Pyrin domain (PYD) a central NOD, and a C-terminal LRR. However, some other members of this numerous subfamily present structural differences, such as NLRP1 and NLRP10. NLRP1 protein includes all the hallmark of NLRP domains, but also, and distinct from other members of the family, a C-terminal Function-to-find domain (FIIND) and a CARD domain that are fundamental for the inflammasome activity (72). On the other hand, NLRP10 is the only member of the NLR family that does not contain an LRR domain (73).

#### Signaling

The NLRP1 inflammasome was the first to be discovered as an intracellular molecular platform that could recruit and activate pro-caspase 1 (74). While the activation mechanisms of NLRP1 are still not completely understood, accumulated evidence indicates that NLRP1 does not work as a MAMP receptor, but instead as a direct sensor of pathogen activities such as proteolysis. Indeed, it has been reported that proteolytic degradation of NLRP1 is necessary and sufficient to induce inflammasome activation (75). While counterintuitive, most reported observations support that a “functional degradation” of the NLRP1 molecule will lead to a CARD-dependent inflammasome formation (75, 76).

NLRP3 is the best-known member of the NLRP subfamily and the best-studied inflammasome so far. The NLRP3 molecular domain structure is the prototypic NLRP (77). Before activation, and similarly to NOD1 and NOD2, NLRP3 is present in an autoinhibited conformation where the LRR domain is folded over the NOD domain (77). The exact mechanisms for NLRP3 activation are still not completely understood but it has been proposed that it is a two-step process that requires two different signals to promote inflammasome assembly (78). The priming step or first signal would be provided by MAMP or DAMP inflammatory stimuli such as TLR4 agonists, which will induce NF-κB-mediated NLRP3 expression. After priming, NLRP3 activation will be promoted by a second signal that can be provided by a wide variety of stimuli, including bacteria-derived molecules such as LPS, viral RNA, fungal hyphae, endogenous DAMP like ATP, or the exposure to environmental irritants including alum (77, 79). The common factor for NLRP3 activators is their ability to induce cellular stress, however, the exact mechanism for NLRP3 sensing of this cellular stress is still not clear. After activation, NLRP3 will adopt an active conformation, that allows the oligomerization and self-association (80). Oligomerized NLRP3 recruits the adaptor protein ASC through homotypic PYD–PYD interaction and nucleates the formation of ASC filaments that can coalesce into a single macromolecular structure denominated ASC speck (81). The formation of these structures allows the recruitment of pro-caspase 1 through CARD–CARD with ASC interactions and enables caspase 1 self-cleavage and activation (82). Activated caspase 1 processes pro-IL1β and pro-IL18 into their mature active forms, which are then secreted into the extracellular space to perform their immune functions (83) (**Figure 2B**).

#### Expression

NLRP1 is widely expressed in many human tissues, including immune cells, the digestive and respiratory epitheliums, or the brain (84, 85). NLRP3 is highly expressed in a variety of innate immune cells including, macrophages, dendritic cells, and neutrophils, and also in T and B lymphocytes. Furthermore, NLRP3 expression has also been detected in intestinal and respiratory epithelial cells (86).

### NLRX

#### Structure

NLRX1, the only described member of the NLRX subfamily, lacks a fully characterized N-terminal domain. However, within this N-terminal region, a mitochondria-targeting sequence (MTS) has been identified (87). NLRX1 C-terminus structure is also unique containing seven LRR domains followed by an uncharacterized three-helix bundle likely involved in molecular recognition (88).

#### Signaling

The range of MAMP and DAMP sensed by NLRX1 is far from clear and the downstream signaling pathways and adaptor proteins are still not known. Despite the lack of mechanistic knowledge, several authors had provided evidence supporting a regulatory role for NLRX1 in a pro-inflammatory signaling context. More specifically, it has been shown that NLRX1 negatively regulates NF-κB and type-I interferon signaling, modulates the production of reactive oxygen species (ROS), participates in autophagy and cell death, and impacts JNK and MAPK pathways (89).

#### Expression

NLRX1 is considered to be ubiquitously expressed in mammalian cells (90).

## NLRs in asthma

A continuously growing body of knowledge has linked several members of different NLR subfamilies with asthma pathogenesis and development including but not limited to CIITA (91) NOD1 (92), NOD2 (93), NLRC4 (94), NLRP1 (95), and NLRP3 (96).

### CIITA

Dysregulation or CIITA loss leads to an array of immune disorders. As a matter of fact, one of the first CIITA descriptions was made in a study that explored the underlying mechanisms of bare lymphocyte syndrome (97), a severe combined immune deficiency that results in susceptibility to severe infections and frequently death in early childhood. This condition results in the absence or very low HLA-DR expression on lymphocytes, with reduced CD4^+^ T-lymphocyte counts leading to an inverted CD4/CD8 ratio (98, 99). Similarly, CIITA knockout in mice leads to a lack of expression of MHCII molecules in antigen-presenting cells and impaired T cell maturation and CD4^+^ T cell-mediated antigen responses (100).

#### The genetic link

CIITA polymorphisms have been associated with several immune-mediated diseases such as multiple sclerosis or lupus erythematosus (91). Interestingly, the CIITA gene is located in 16p13.13, a locus that has been associated with asthma and allergy in different GWAS studies, including a metanalysis by Demenais et al. that included 66 genome-wide association studies accounting for 23,948 asthmatic and 118,538 controls from ethnically-diverse origins (101). However, the mentioned locus includes a gene complex that involves other genes such as CLEC16A with several known polymorphisms previously associated with asthma that have been further confirmed in others studies (102). Bae et al. investigated the association between CIITA polymorphisms and the presence of nasal polyps in asthmatic patients in a case-control study of genetic association analysis using blood isolated genomic DNA from 467 asthmatics. This study showed that at least two CIITA SNPs (rs12932187 and rs11074938) and 2 haplotypes (CIITA_BL1_ ht2 and CIITA_BL1_ht5) were associated with nasal polyps development in asthmatics (91) (**Table 1**).

**Table 1:** Most relevant NLR-associated polymorphisms in asthma.

NLR	Polymorphism	Cohort	Asthma association	Author, Year
**CIITA**	rs12932187 and rs11074938	Korean asthmatic adults	Nasal polyp development in asthmatics	Bae et al. (2013) (91)
**NOD1**	ND1 +32656(*2)	Australia & UK families/German asthmatic children	Asthma susceptibility and elevated IgE	Hysi et al. (2005) (92)
	CARD4/-21596T	German children farmers/non farmers	Increased farm environment asthma protective effect	Eder et al. (2006) (103)
	rs2907748, rs2907749 and rs2075822	German atopic adults	Elevated IgE	Weidinger et al. (2005) (93)
	rs2075820A	Tunisian asthmatic children	Asthma susceptibility	Belhaj et al. (2019) (104)
**NOD2**	C2104T, G2722C, 3020iC	German children	Atopy	Kabesch et al. (2003) (105)
	rs1077861 and rs3135500	German asthmatic adults	Asthma susceptibility	Weidinger et al., (2005) (106)
	rs3135499	Chinese asthmatic children	Asthma susceptibility	Cai et al. (2019) (107)
**NLRP1**	rs11651270, rs12150220, and rs2670660	Brazilian asthmatic children	Asthma susceptibility and elevated IgE	Leal et al. (2018) (95)
	rs11651270	Mexican American asthmatic children	Asthma susceptibility	Moecking et al. (2021) (108)
**NLRP3**	rs4612666	Japanese allergic children	Aspirin induced asthma	Hitomi et al. (2009) (96)
	rs10754558	Brazilian asthmatic children	Asthma susceptibility	Leal et al. (2018) (95)
	rs72553860, rs12137901, and rs4925648	Brazilian asthmatic children	Asthma susceptibility	Queiroz et al. (2020) (109)

* used in the polymorphism nomenclature as a separator to indicate the allele.

As previously stated, CIITA acts as a master regulator for MHC II molecules regulating their activity at the level of transcription, therefore CIITA plays a relevant role in the development of adaptive immune responses. However, little is known about the potential implication of CIITA in asthma.

### NOD1 and NOD2

NOD1 and NOD2 are the best-known representatives of the NLRC subfamily. As previously mentioned, these receptors recognize specific bacterial PGN motifs and their activation leads to the recruitment and signal transduction through RIPK2 and activation of different downstream signaling pathways including MAP kinases and NF-κB. In addition to the role of NOD1 and NOD2 in the development of innate immune responses to bacterial and viral infection, both receptors have been implicated in the priming of adaptive immune responses.

#### The genetic link

The NOD1 gene is located in the 7q14-p15 chromosome region, a locus previously identified by different genome-wide association analyses as an atopy and asthma-related trait susceptibility locus (110, 111). As part of a study of candidate genes in this locus, Hysi et al. identified NOD1 as a relevant gene for asthma development. A systematic search of polymorphic alleles in DNA obtained from blood, described for the first time NOD1 polymorphisms associated with asthma and high levels of seric IgE. ND1+32656*2 was identified as the strongest associated polymorphism (92). Moreover, exploring the role of the farming environment on the development of allergic asthma, Eder et al. using NOD1 haplotype tagging SNPs in DNA obtained from children’s blood samples, found that 21596 T SNP in NOD1 can drastically modify the effect of environmental factors in asthma, correlating with higher frequencies of atopic asthma symptoms (103). After these first genetic studies, several authors have confirmed the link between NOD1 gene polymorphisms and haplotypic combinations with atopy and asthma development in different populations. By SNP genotyping in a large German adult cohort of atopic patients, Cai Weidinger et al. identified the association of NOD1 SNPs rs2907748, rs2907749, and rs2075822 with IgE levels (106). More recently, Belhaj et al. found an association between the NOD1 rs2075820 variant and childhood asthma in a Tunisian cohort (104). Most likely, these polymorphisms may affect bacterial sensing and hence modify the downstream signaling pathways that normally would be triggered by NOD1 (45).

Similarly, different authors have reported results supporting the existence of a genetic link between the polymorphisms in the NOD2 gene and asthma development. Kabesh et al. showed that NOD2 polymorphisms are linked to atopy in children, however, they failed to find significant direct associations with asthma in a German children cohort (105). Weidinger et al. provided more data supporting the implication of NOD2 polymorphisms in respiratory atopy in an adult cohort, and importantly, showed a significant association of rs1077861 and rs3135500 with asthma development (93). Following these studies, other authors have explored the role of NOD2 polymorphisms in asthma predisposition in different cohorts reporting conflicting results. While some have failed to show any significant association between NOD2 genetic polymorphisms and asthma (104), more recent studies have provided data backing up the existence of a link between NOD2 polymorphisms and asthma. For example, Cai et al. published a Chinese cohort study, showing an association of the rs3135499 C allele and increased asthma development (107).

#### Human samples and *in vitro* studies

##### Airway epithelial cells

As mentioned before, NOD1 and NOD2 are both expressed and can be functionally active in human bronchial epithelial cells (62, 112). Despite this fact, the reported results seem to indicate that there are differences in the implication of NOD1 and NOD2 in the lung epithelium in the context of asthma. It has been shown that the stimulation of human bronchial epithelial cells with a NOD1 agonist induces the release of IL8 and CCL2 among other down stream mediators, while NOD2 activation does not result in changes in either IL8 or CCL2 production (113). Accordingly, although, our group has shown that the stimulation of human bronchial epithelial cells with HDM induces NOD1 and NOD2 gene expression and IL8 production (114), only NOD1 knockdown in human bronchial epithelial cells reduced significantly IL8 production (114). NOD2 (as well as NLRC5) gene expression has also been shown to be increased in influenza-stimulated primary bronchial epithelial cells from asthmatic patients (115).

##### Airway smooth muscle cells

These cells are also relevant effector cells in asthma by affecting AHR, and airway remodeling (116). As is the case in the bronchial epithelium, both NOD1 and NOD2 expression have been detected in the smooth muscle cells of the airway (63, 117). Kvarnhammar et al. reported that while both NOD1 and NOD2 gene expression was detectable on *in vitro* cultured human airway smooth muscle cells, NOD2 was not detectable at a protein level by flow cytometry or immunocytochemistry. Moreover, the stimulation of airway smooth muscle cells with a NOD1 agonist, but not with a NOD2 agonist, significantly increased the production of IL8 and IL6 (63). In contrast, and suggesting that both NOD1 and NOD2 play a role in airway smooth muscle cells in asthma, Ni et al. showed that NOD2 was upregulated in the smooth muscle cells of asthmatics after stimulation with a NOD2 ligand and that this stimulation resulted in increased IL6 and Thymic Stromal Lymphopoietin (TSLP) production (117). Nonetheless, these contradictory results could be related to the different transcriptional mechanisms governing NOD1 and NOD2 expression. Indeed, NOD1 and NOD2 are under the control of different transcription factors, namely NFAT (118) for the former and NF-κB (119) for the latter. Thus, the expression of NOD2 may only be present and detected in inflammatory conditions.

##### Eosinophils and neutrophils

It has been reported that human eosinophils can be activated *in vitro* by NOD1 or NOD2 agonists (120, 121) and that this activation can promote their migration and interaction with bronchial epithelial cells (113, 121). Moreover, the stimulation with NOD1 and NOD2 ligands of a coculture of eosinophils and human bronchial epithelial cells resulted in enhanced concentrations of IL8 and CCL2 (113). As eosinophils, neutrophils play a very important role in asthma, especially in some of the most severe forms. As stated previously, NOD1 and NOD2 activation in different human cells, such as human bronchial epithelial, or airway smooth muscle cells leads to the production of pro-inflammatory cytokines including IL8 which is implicated in the recruitment of neutrophils to the inflammation sites (44). Moreover, neutrophils are not passive players in the response through NOD1 and NOD2 as they also express both these receptors (122, 123) and it has been reported that *in vitro* stimulation of human neutrophils with NOD2 agonists results in the production of IL8 in a dose-dependent manner.

##### Monocytes, macrophages and dendritic cells

The presence of NOD1 and NOD2 expression in human monocytes, macrophages and dendritic cells has been well documented since their discovery and first descriptions (28, 29). Early studies confirmed that the stimulation of monocytes, macrophages and dendritic cells with NOD1 and NOD2 agonists results in the production of pro-inflammatory cytokines such as IL1β, IL6, IL8, as well as promoting the maturation of these cells (124). In agreement with these previous findings but in the context of asthma, our group has shown that *in vitro* stimulation of human dendritic cells obtained from asthmatics with a NOD1 ligand results in the production of IL6 and IL8, and interestingly enough of the pro-Th2 chemokines CCL17 and CCL22 (125). Importantly, we have also shown that NOD1 ligand primed human dendritic cells induce the polarization of T cells into Th2 cells with elevated production of IL13 (125).

##### T cells

The innate immune sensing of PGN moieties by NOD1 has been shown to be a relevant player in the priming of antigen-specific T cell and B cells responses *in vivo*. The stimulation of the NOD1 receptor was shown to be sufficient to drive the polarization of specific Th2 responses (126). Similarly, it has been reported that the stimulation of NOD2 elicits antigen-specific Th2 polarization and immune responses characterized by the production of the type 2 cytokines IL4 and IL5, and also the production of IgG1 (127). Furthermore, additional mechanisms and cells seem to be implicated in NOD1 and NOD2 dependent development of Th2 responses including the induction of OX40 ligand on dendritic cells through the production of TSLP by structural cells (128). Furthermore, it has been described that NOD2 can have a T cell intrinsic role in the generation of effective T helper responses. Shaw et al. showed that T cell differentiation into Th1 and Th2 is severely impaired in NOD2 deficient T cells due to a decreased production of IL2 (129). More recently, Napier et al. demonstrated an unusual role for NOD2, modulating Th17 responses downstream of T cell receptor and CD28 activation, independently of RIPK2 (130).

#### *In vivo* studies

Different experimental models have been used to explore and confirm the findings obtained in human samples and *in vitro* studies regarding the implication of NOD1 and NOD2 in different aspects related to asthma pathophysiology. Wong et al. reported that the intravascular administration of NOD1 or NOD2 ligands to mice subjected concomitantly to an OVA and alum asthma model induced significant increases in bronchoalveolar lavage (BAL) eosinophils, BAL levels of IL13, and seric total IgE (113). However, as exemplified by the Wong et al. study, most of the studies were based on non-physiological routes of administration and adjuvants.

In agreement with the genetic link to asthma and the adaptive immune modulation role of NOD1, our group has reported that a NOD1 agonist can exert adjuvant effects exacerbating the asthmatic response in an OVA-induced Th2-mediated allergic model. Importantly, the administration of the NOD1 ligand promoted significant changes in all the hallmarks of asthma models including increased AHR and eosinophil recruitment, higher levels of seric specific IgE and IL13 production among other asthma-related mediators (125). Moreover, the mechanisms by which the concurrent NOD1 stimulation in this OVA-induced model aggravated asthma were related to the higher activity of dendritic cells and increased production of the pro-Th2 chemokine CCL17 (125).

RIPK2 is the downstream adaptive protein in both the NOD1 and NOD2 signaling pathways, and as such, is a critical mediator in the NOD1 and NOD2-related implication in asthma development. Polymorphisms in RIPK2 have been related to some cases of severe childhood asthma (131). Goh et al. showed using an OVA murine model that not only the protein levels of RIPK2 are increased in asthma, but also, and more importantly, that the knockdown of RIPK2 *via* intratracheal administration of a specific siRNA ameliorates the experimental asthma phenotype including significant reductions in AHR, eosinophil recruitment, lower levels of IL4, IL5, IL13, IL33 and seric OVA-specific IgE (132). Despite the solid results provided by the RIPK2 knockdown, other authors have failed to find any significant differences between RIPK2 deficient mice and the wild type (WT) counterparts when subjected to an OVA-induced asthma protocol (133). However, using a much more physiologically relevant model based on HDM, Miller et al. showed that RIPK2 deficient mice exhibited a reduction in the eosinophil recruitment, the production of Th2 and Th17 cytokines, and the levels of HDM-specific IgG1 (134). Furthermore, the histological analysis of the RIPK2 deficient mice lungs showed an improvement of the classic lung pathology features of asthma.

In accordance with these results, our group has shown that both NOD1 deficient and RIPK2 deficient mice, when subjected to an HDM asthma protocol, present reduced asthma features including reduced AHR, eosinophil, and neutrophil recruitment. In agreement, the levels of IL5, IL13, and IL33 among other cytokines, coincided with reduced asthma histopathology features in their lungs, indicating that NOD1 aggravates HDM-induced asthma through RIPK2 (114). Similar to the NOD1 deficient mice, the NOD2 deficient mice displayed decreased eosinophil and neutrophil recruitment. However, there were no differences in AHR, Th2 cytokine production, or any of the other murine model asthma hallmark features. Importantly, we have also shown using NOD1 deficient bone marrow transplants that epithelial cells play a more relevant role in the reduced asthmatic response in the NOD1 deficient mice than the bone marrow derived cell subsets. Moreover, the HDM *in vitro* stimulation of NOD1 knockdown human bronchial epithelial cells further supported the higher relevance of NOD1 compared with NOD2, in the aggravation of asthma most likely *via* the differential expression of agonist transporters at the respiratory epithelium (114).

Most importantly, our group has also shown *via* microbiota transplantation experiments that the aforementioned NOD1-related HDM asthma aggravation mechanisms are gut microbiota-independent. Indeed, despite the NOD1 deficient mice presenting dysbiotic gut microbiota, the transplantation of the NOD1 deficient microbiota into WT mice did not result in any significant changes in the HDM experimental asthma model outcome. Notwithstanding, we showed that not only it was possible to detect NOD1 specific ligands in HDM extracts, but also that the administration of PGN depleted HDM extracts to WT mice reproduced the NOD1 deficient mice phenotype. These findings indicate that the sensing of NOD1 specific ligands present in HDM extracts is most likely the underlying cause of the NOD1 linked HDM asthma aggravation. It highlights an unprecedented interaction between NOD1 and HDM, unveiling a new mechanism whereby HDM-derived microbiota potentiates disease severity through NOD1 and RIPK2 signaling (114).

All these aforementioned findings, and others such as the synergy of NOD1 and NOD2 signaling with the actions of TLR receptors in the priming of Th2 and Th17 immune responses (126), directly implicate NOD1 and NOD2 and their signaling pathways in several mechanisms and cells related to asthma development and exacerbation.

### NLRC4

#### The genetic link

A transcriptomic study using induced sputum from severe and mild asthmatics showed that NLRC4 is upregulated in patients with neutrophilic and mixed granulocytic cell profiles in induced sputum. However, no significant differences were found between mild and severe asthma (94).

#### *In vivo* studies

Using three different models of HDM-induced asthma designed to represent eosinophilic, mixed, and neutrophilic acute asthma phenotypes Tan et al. found that in all of them, there was an increased expression of NLRC4 mRNA, with the neutrophilic model presenting the highest expression (135).

Hitherto, data availability is yet insufficient to assess a functional role of NLRC4 in asthma and awaits further evaluation.

### NLRP1

#### The genetic link

Leal et al. showed in a Brazilian asthmatic children cohort that three NLRP1 SNPs rs11651270, rs12150220, and rs2670660 were associated with asthma in a family association study. In the same cohort, minor alleles of two of the NLRP1 SNPs (rs11651270/C and rs2670660/G) also showed an association to asthma severity and high levels of IgE (95).

Similarly, Moecking et al. explored the relationship between NLRP1 and asthma in a Mexican American asthmatic children cohort to find that the NLRP1 2A haplotype was associated with asthma (108). Using single-variant association testing they found that, as it was the case in the Brazilian cohort, the rs11651270 NLRP1 SNP was associated with asthma. In particular, increased copies of the C allele of rs11651270 were associated with increased asthma susceptibility. Interestingly, rs11651270 results in an amino acid substitution from Methionine 1184 to Valine (M1184V) that has been described to increase cleavage in the FIIND domain that can result in alterations of NLRP1 activation (72). Despite the concordance in the correlation of rs11651270/C between the Brazilian and Mexican American children cohorts, further attempts of validation failed in providing confirmation for the effect of these NLRP1 SNPs in Puerto Rican and African American cohorts (108).

#### *In vivo* studies

Moecking et al. also explored the potential role of NLRP1 in a murine model of experimental asthma. While an NLRP1 deficient mouse did not show asthma features at baseline when compared to a WT control, an OVA+Alum asthma experimental model resulted in increased eosinophil infiltration to the lungs of the NLRP1 deficient mice. Furthermore, the NLRP1 deficient mice showed increased IL13 levels in BAL, suggesting a protective effect for NLRP1 in the context of asthma. Using IL1R and IL18 deficient mice and the same asthma model, it was found that the potential protective effect of NLRP1 was not related to IL1R signaling, but to IL18 since IL1R but not IL18 deficient mouse, failed to reproduce the NLRP1 deficient phenotype (108).

Taken together these data provide evidence for an NLRP1 dependent IL18-mediated protective role in asthma, that could be disrupted by an altered NLRP1 activation related to polymorphisms such as rs11651270/C.

### NLRP3

The NLRP3 inflammasome has been shown to participate in the pathogenesis of many diseases with an inflammatory component. However, the implication of NLRP3 in asthma has been a matter of controversy.

#### The genetic link

NLRP3 polymorphisms have been significantly associated with the susceptibility to develop asthma in different cohort studies. Hitomi et al. showed in a pediatric cohort using DNA obtained from blood that NLRP3 rs4612666 was significantly associated with aspirin-induced asthma (96). Leal et al. also identified in their asthmatic children cohort NLRP3 rs10754558 as a relevant polymorphism in asthma (95). Another recent study conducted using blood samples from an asthmatic children cohort found that the G, C, and T alleles of rs72553860, rs12137901, and rs4925648 NLRP3 SNPs were also associated with asthma susceptibility (109).

#### Human samples and *in vitro* studies

NLRP3 expression has been described in different human cell subsets that could potentially play relevant roles in asthma pathogenesis (136).

##### Airway epithelial cells

It has been shown that NLRP3 is expressed in the bronchial epithelium of healthy subjects at baseline (137). Interestingly, stimulation of human bronchial epithelial cells with a major allergen of house dust mite Der f 1, induced the release of IL-1β through NLRP3 activation as shown by RNA silencing (138). Although not entirely related to epithelial cells, BAL fluid from asthmatic patients at baseline exhibited higher NLRP3 protein content than healthy controls (139), suggesting a role for NLRP3 in asthma.

##### Neutrophils

Noteworthy, the expression of NLRP3 is increased in the sputum of severe T2 low neutrophilic asthma. The levels of NLRP3 mRNA in steroid resistant asthmatics correlate with the numbers of neutrophils, and the severity of asthma (140), while levels of IL-1 β correlate with IL-8, a neutrophil attracting chemokine (141), suggesting a role of NLRP3 in the neutrophil asthma phenotype. Indeed, in the latter study, they evidenced by immunostaining neutrophils expressing NLRP3 in this subset of asthma patients (141).

##### Macrophages

As resident myeloid cells in the lung, alveolar macrophages are an obvious candidate where NLRP3 could play a role in the development of asthma. Sputum macrophages have been shown to express NLRP3 in neutrophilic asthma (141). Moreover, Gordon et al. have recently reported that the NLPR3 inflammasome can be activated in BAL-derived macrophages from asthmatic subjects inducing the secretion of IL1β (142). However, as for the other cells, the precise implication of macrophages remains elusive.

##### T cells

Importantly, although most of the attention regarding a potential role for NLRP3 in asthma has been directed to myeloid cells and the lung epithelium, the relevance of NLRP3 in the asthma context is not restricted to neutrophilic responses and phenotypes. Indeed, it has been reported that NLRP3 expression is significantly increased in severe asthma patients where it could be participating in predominantly Th2 responses (94). Additionally, it has been found that lymphoid cells and more specifically CD4^+^ T cells are able to express NLRP3 (143). Furthermore, NLRP3 can be activated in CD4^+^ T cells with subsequent NLRP3 inflammasome formation and IL1β production. Some authors have explored the role of NLRP3 in CD4^+^ T cells to find not only that NLRP3 is induced upon T cell receptor activation, but interestingly that NLRP3 could play a role in the differentiation of Th2 cells in an inflammasome-independent manner cooperating with the transcription factor IRF4 (144).

#### *In vivo* studies

Contradictory *in vivo* studies have been published with some providing inconclusive results regarding a relevant implication of NLRP3 in asthma, and others providing support for a prominent role for NLRP3 in asthma.

Among the negative studies, Kool et al. reported that the Th2 asthmatic airway inflammation generated in response to a combination of OVA and alum developed normally in Nlrp3 deficient mice (145). Moreover, Allen et al. using alum-free OVA and HDM models reported that NLRP3 deficient mice presented a similar phenotype to the WT mice when subjected to the experimental asthma protocols. However, while most of the asthma model hallmarks such as AHR did not present significant changes between the NLRP3 deficient mice and the WT, the levels of IL13 and IL33 were slightly but significantly attenuated (146). In a model of HDM-induced asthma, NLRP3 inflammasome even dampened Th2 responses through caspase-1 activation (147). These studies being the cause of controversy in the field, suggest that NLRP3 seems to play only a minor role in asthma development.

In contrast, Hirota et al. showed that NLRP3-dependent responses can influence adaptive responses through the production of IL1β, promoting airway neutrophilia and increasing dendritic cells numbers (137). Kim et al. using a slightly different approach using respiratory infection models and OVA to induce severe steroid-resistant allergic asthma found that the steroid-resistant neutrophilic inflammation and increased AHR were driven by increases in NLRP3, caspase-1, and IL1β responses (140). Using an OVA and alum-induced allergic asthma model, Eisenbarth et al. showed that NLRP3 deficient mice presented reduced pulmonary inflammation with decreased eosinophilic recruitment, lower levels of IL5, and a decrease in OVA-specific IgG1 production (148). In addition to this, the NLRP3 role in priming Th2 responses was further demonstrated in an OVA model without any adjuvant. Besnard et al. showed that NLRP3 deficient mice subjected to OVA challenges not only presented a reduction in the eosinophil recruitment, but also, significantly lower levels of TSLP, IL33, IL5, and reduced serum levels of OVA-specific IgE. Moreover, the deficiency in NLRP3 resulted also in reduced expression of IL4, IL13, and CCL17 and a reduction in the migration of dendritic cells to the lymph nodes (149). In addition, some authors have provided data that implicates the NLRP3 response in Th17 responses in asthma (150), proposing an NLRP3-IL1 β -Th17 signaling axis (151).

Based on the previously described findings and the broad spectrum of asthma mechanisms with potential NLRP3 participation, some authors have tried to explore the implication of NLRP3 in different asthma endotypes. Recently, Tan et al. compared the role of NLRP3 in three different models of HDM-induced asthma designed to represent eosinophilic, mixed, and neutrophilic acute asthma phenotypes (135). It was found that each of these models displays significant differences in the expression of NLRP3 and NLRP3 activation related genes. However, while the mixed and neutrophilic models exhibited an increased expression of NLRP3, the adaptor molecule ASC and pro-IL1β, the eosinophilic model expressed significantly higher levels of ASC but no significant upregulation of the other components of the NLRP3-mediated response.

Moreover, and in accordance with previous findings, Bruchard et al. showed that NLRP3 deficient mice subjected to an OVA asthma experimental protocol showed reduced eosinophil and lymphocyte recruitment into the lungs as well as higher accumulation of mucus and reduced concentration of IL4 and IL5. Most interestingly, the involvement of NLRP3 in CD4^+^ T cells in asthma, was elegantly confirmed by the restoration of the asthmatic phenotype in NLRP3 deficient mice by transferring WT OVA-specific Th2 cells (144).

Despite all the evidence supporting the NLRP3 implication in asthma the underlying mechanisms of NLRP3 activation are still far from being completely understood. Kim et al., based on human expression data and OVA and HDM murine models, presented evidence supporting a critical role for mitochondrial ROS in the pathogenesis of asthma through the modulation of NLRP3 activation (139). Further supporting a role for mitochondrial ROS in the activation of NLRP3 in the asthma context, Sebag et al. provided evidence indicating that a mitochondrial Ca2+/calmodulin-dependent protein kinase II mediates mitochondrial ROS production, which stimulates NLRP3 inflammasome activation in the airway epithelium and promotes asthma development (152).

Altogether, although still controversial, these data suggest that NLRP3 may play a role in both T2 low neutrophilic and T2 high eosinophilic severe asthma.

## Therapeutic targeting of NLR and asthma

The accumulation of evidence supporting a role for NLR as critical regulators of asthma development has spurred the study of different strategies to target NLR pathways as an alternative or a complement for conventional asthma treatments (**Table 2**).

**Table 2:** Compounds targeting the NLR pathway and their effects in asthma models.

Target	Compound/molecule	Effect on target	Administration route	Asthma Model	Effects on asthma	Author, Year
RIPK2	GSK583	Inhibition	Oral/Diet	HDM	Reduction in eosinophilia, neutrophilia, Th2, and Th17	Miller et al. (2020) (153)
NLRP3	MCC950	Inhibition	Oral	HDM	Reduction in neutrophilia, CXCL1, and CXCL5	Primiano et al. (2016) (154)
			Intraperitoneal	OVA/infection steroid resistant murine model	Reduction in neutrophilia, and AHR	Kim et al. (2017) (140)
			Intraperitoneal	OVA + Alum	Reduction in eosinophilia, neutrophilia, IL4, IL13	Wang et al. (2018) (155)
			Intraperitoneal	OVA + Alum	Reduction in TSLP, CCL2	Lv et al. (2018) (156)
			Intraperitoneal	Toluene diisocyanate	Reduction in eosinophilia, neutrophilia Th2, Th17, and AHR	Chen et al. (2019) (157)
NLRP3	OLT1177 (dapansutrile)	Inhibition	Intraperitoneal	OVA	Reduction in eosinophilia, AHR, IL4, IL5, IL13	Lunding et al. (2021) (158)
			Intraperitoneal	HDM	Reduction in eosinophilia, neutrophilia, and AHR	Lunding et al. (2021) (158)
			Oral/Diet	OVA	Reduction in eosinophilia, and AHR	Lunding et al. (2021) (158)
NLRP3	Suhuang (YBZ00172008)	indirect Inhibition?	Intragastric	OVA + Alum (Rat)	Reduction in neutrophilia, and IgE	Qin et al. (2019) (159)
NLRP3	Yupingfeng San	indirect Inhibition?	Intraperitoneal	OVA + NaOH	Reduction in eosinophilia, and neutrophilia	Liu et al. (2017) (160)
NLRP3	Dexamethasone	indirect Inhibition?	Intraperitoneal	OVA + Alum	Reduction in eosinophilia, neutrophilia, IL5 and IL17	Guan et al. (2020) (161)

### NOD1 and NOD2

The relevant role of NOD1 and NOD2 not only in asthma but also in other diseases with an inflammatory component, such as Crohn´s disease (162), has encouraged the development of different compounds with the ability to target their signaling pathways, mainly the adaptor protein RIPK2. RIPK2 inhibitors have already shown that they are not only effective in blocking NOD1 and NOD2 signaling (163, 164), but also their efficacy in modulating human inflammatory responses (165, 166). Indeed, very promising highly selective, and potent RIPK2 inhibitors, that even have reached the clinical trial phase have been already developed (164). However, most of the inhibitors already tested have been relegated as tool compounds due to less than optimal pharmacokinetic profiles (167). Ongoing development of improved inhibitors and of new compounds to overcome these limitations would represent potential future tools for asthma modulation. Accordingly, Miller et al. have explored the use of one of these promising RIPK2 inhibitors in an HDM experimental asthma model with relative success (153). The administration of the selective GSK583 RIPK2 inhibitor *via* the mice diet prior to and during the HDM sensitization phase of an acute asthma model resulted in a reduction of the eosinophilia, neutrophilia, and histopathology features of the asthmatic mouse. Moreover, this preventive RIPK2 inhibition also reduced the Th2 and Th17 cell recruitment and the levels of IL4 and IL5. Despite this adaptive response modulation, RIPK2 inhibition did not result in changes in AHR or the humoral response (153). The relative success in reducing experimental HDM asthmatic features even in a “preventive” approach, provides a solid rationale for a potential future therapeutic application of the NOD1 and NOD2 signaling targeting through RIPK2 inhibition.

### NLRP3

As mentioned previously NLRP3 inflammasome has been implicated in a diverse array of diseases. This fact has fueled the development and characterization of molecules that could inhibit NLRP3 or other inflammasome components in order to understand the molecular mechanisms implicated in disease and their potential therapeutic applications. Several NLRP3 inhibitors have been reported to date, including those that either directly or indirectly inhibit NLRP3 inflammasome or related signaling events. However, the inhibitory mechanism is not always well-characterized or the precise targets are not fully elucidated. With the accumulation of evidence supporting the implication of NLRP3 in asthma development, some authors had started to consider and test these inhibitors as potential asthma therapeutic tools.

One of the best characterized and the most potent of NLRP3 inhibitors is MCC950, first described by Perregaux et al. as a compound capable of inhibiting LPS and ATP induction of IL1β (168). MCC950 binds directly and specifically to NLRP3 irrespective of its activation state, impairing conformational rearrangements, blocking NLRP3 in an inactive conformation, and preventing inflammasome assembly (169, 170). Primiano et al. showed that oral administration of MCC950 prior to sensitization in an acute HDM-induced asthma model, not only efficiently inhibited NLRP3 and reduced IL1β levels, but importantly, also blocked asthma development and reduced the levels of Th2 cytokines and eosinophil and neutrophil recruitment (154). In a steroid-resistant severe asthma model, Kim et al. found that MCC950 treatment, even at low doses, was capable of suppressing neutrophil recruitment and reducing AHR (140). The efficacy of MCC950 has also been reported in a model of chemically-induced asthma in mice. The administration of MCC950 to a toluene diisocyanate-induced asthma model blocked the activation of NLRP3 and downregulated protein expression of caspase-1, IL1β, and IL18. Furthermore, NLRP3 inhibition resulted in AHR reduction, decrease of asthma histopathological features, and suppression of Th2 and Th17 responses (157). Additionally, other authors have also provided evidence for the efficacy of MCC950 in the classic OVA-induced asthma model (155, 156). Recently, another specific NLRP3 inhibitor was tested in a murine model of asthma with promising results. Lunding et al. reported that the administration *via* enriched mouse diet of OLT1177, a specific NLRP3 inhibitor shown to be safe in humans (171), reduced AHR, eosinophil, and neutrophil numbers in BAL and inflammatory infiltrate in the lung in an OVA asthma model (158).

Interestingly, some compounds already in use for the treatment of asthma or to attenuate airway inflammation have been found to be effective at least partially because they are able to inhibit the NLRP3 inflammasome. Among these compounds with NLRP3 inflammasome suppressing capabilities, there are several medicinal plants and natural products, such as the *Hibiscus noldeae* (172), Suhuang (159), or the Yupingfeng San (160) whose efficacy through NLRP3 have been unveiled in murine models. The most relevant of the previously known effective compounds is however dexamethasone. Guan et al. recently reported using an OVA-induced murine model that dexamethasone, alleviates allergic airway inflammation partially by inhibiting NLRP3 inflammasome and reducing IL1β and IL18 levels (161).

Alternatively, other authors have targeted the NLRP3 inflammasome downstream signaling, and more specifically some of them have provided promising evidence targeting IL1β. Ritter et al. reported that the administration of Anakinra, a non-glycosylated recombinant form of the naturally occurring IL1 receptor antagonist (IL1RA), reduced asthma development in an OVA asthma model (173). Moreover, in a study with healthy volunteers, pretreatment with Anakinra significantly diminished IL1β, IL6, IL8, and airway neutrophilia induced by LPS nasal challenge (174). These results and others pointing to the potential efficacy of targeting IL1β have led to the development of alternative agents such as rilonacept (IL1 trap) (175) and canakinumab (anti-IL1β antibody) (176) that could also be potential tools for IL1β modulation in asthma.

## Conclusions

NOD-like receptors are master regulators of innate responses and their signaling pathways in different cell types play pivotal roles in shaping adaptive immunity. In the context of asthma, although many NLR functions and mechanisms are still unknown, it is now clear that at least some of them, namely NOD1, NOD2, and NLRP3, play crucial roles in the development, regulation, and exacerbation of asthma. However, despite the well documented genetic association of both NOD1 and NOD2 with asthma development, and the wealth of information about the implication of these two receptors in other relevant mechanisms implicated in this prevalent disease, a detailed understanding of the specific mechanisms implicated in the development or exacerbation of asthma *via* NOD1 and NOD2 are still not completely understood. A myriad of reports have supported the implication of NLRP3, however as is the case with the aforementioned NOD1 and NOD2, the complete understanding of the underlying mechanisms is still far from our reach. Moreover, in the case of NLRP3, the existence of contrasting findings further pinpoints the need for a deeper understanding of asthma-related NLRP3 and inflammasome-driven mechanism, and at the same time, underlines the complexities of asthma. Notwithstanding, the available data and knowledge about the implication of NLRs in asthma make them very interesting targets for the development of complementary or standalone disease-modifying treatments.

As a matter of fact, most current available asthma treatments, both classic and biologic, while being safe and very effective in some patients are designed to target asthmatic symptoms, with the exception of allergen immunotherapy that can only be used in a relatively small patient subset. Therefore, the development of new therapies based on the modulation or inhibition of NLRs could represent a leap in asthma treatment potentially providing new alternatives to the already established and available treatments and representing a new disease-modifying alternative by targeting innate and adaptive upstream events.

The available data about the NLR implication in asthma opens new perspectives not only in the understanding of the mechanisms of asthma but also for the future development of new alternative preventive and therapeutic treatments for this prevalent disease.

## Data availability statement

The original contributions presented in the study are included in the article/supplementary material. Further inquiries can be directed to the corresponding author.

## Author contributions

DAS and AT drafted the first manuscript. All listed authors made a significant direct intellectual contribution tothe work and approved it for publication.

## Funding

The work was supported by grants from PTR (18-16) from Institut Pasteur (to AT), by ANR 18-CE14-0020 (to AT, MC and IB).

## Conflict of interest

The authors declare that the research was conducted in the absence of any commercial or financial relationships that could be construed as a potential conflict of interest.

## Publisher’s note

All claims expressed in this article are solely those of the authors and do not necessarily represent those of their affiliated organizations, or those of the publisher, the editors and the reviewers. Any product that may be evaluated in this article, or claim that may be made by its manufacturer, is not guaranteed or endorsed by the publisher.

## References

[1] Dharmage SC Perret JL Custovic A . Epidemiology of asthma in children and adults. Front Pediatr (2019) 7:246. doi: 10.3389/fped.2019.00246 31275909 PMC6591438

[2] Lambrecht BN Hammad H Fahy JV . The cytokines of asthma. Immunity (2019) 50:975–91. doi: 10.1016/j.immuni.2019.03.018 30995510

[3] Jacquet A . Characterization of innate immune responses to house dust mite allergens: Pitfalls and limitations. Front Allergy (2021) 2:662378. doi: 10.3389/falgy.2021.662378 35386970 PMC8974781

[4] Pivniouk V Gimenes Junior JA Honeker LK Vercelli D . The role of innate immunity in asthma development and protection: Lessons from the environment. Clin Exp Allergy (2020) 50(3):282–90. doi: 10.1111/cea.13508 31581343

[5] Hammad H Lambrecht BN . The basic immunology of asthma. Cell (2021) 184(6):1469–85. doi: 10.1016/j.cell.2021.02.016 33711259

[6] Janeway CA . Approaching the asymptote? evolution and revolution in immunology. Cold Spring Harb Symp Quant Biol (1989) 54:1–13. doi: 10.1101/sqb.1989.054.01.003 2700931

[7] Li D Wu M . Pattern recognition receptors in health and diseases. Signal Transduct. Target Ther (2021) 6:1–24. doi: 10.1038/s41392-021-00687-0 34344870 PMC8333067

[8] Accolla RS Jotterand-Bellomo M Scarpellino L Maffei A Carra G Guardiola J . aIr-1, a newly found locus on mouse chromosome 16 encoding a trans-acting activator factor for MHC class II gene expression. J Exp Med (1986) 164(1):369–74. doi: 10.1084/jem.164.1.369 PMC2188193 3088202

[9] Accolla RS Ramia E Tedeschi A Forlani G . CIITA-driven MHC class II expressing tumor cells as antigen presenting cell performers: Toward the construction of an optimal anti-tumor vaccine. Front Immunol (2019) 10:1806. doi: 10.3389/fimmu.2019.01806 31417570 PMC6682709

[10] Nickerson K Sisk TJ Inohara N Yee CSK Kennell J Cho MC . Dendritic cell-specific MHC class II transactivator contains a caspase recruitment domain that confers potent transactivation activity. J Biol Chem (2001) 276:19089–93. doi: 10.1074/jbc.M101295200 11279191

[11] León Machado JA Steimle V . The mhc class ii transactivator ciita: Not (quite) the odd-one-out anymore among nlr proteins. Int J Mol Sci (2021) 22:1–19. doi: 10.3390/ijms22031074 PMC7866136 33499042

[12] Linhoff MW Harton JA Cressman DE Martin BK Ting JP-Y . Two distinct domains within CIITA mediate self-association: Involvement of the GTP-binding and leucine-rich repeat domains. Mol Cell Biol (2001) 21:3001–11. doi: 10.1128/mcb.21.9.3001-3011.2001 PMC86929 11287606

[13] Silacci P Mottet A Steimle V Reith W Mach B . Developmental extinction of major histocompatibility complex class II gene expression in plasmocytes is mediated by silencing of the transactivator gene CIITA. J Exp Med (1994) 180:1329–36. doi: 10.1084/jem.180.4.1329 PMC2191691 7931066

[14] Muhlethaler-Mottet A Otten LA Steimle V Mach B . Expression of MHC class II molecules in different cellular and functional compartments is controlled by differential usage of multiple promoters of the transactivator CIITA. EMBO J (1997) 16:2851–60. doi: 10.1093/emboj/16.10.2851 PMC1169893 9184229

[15] Piskurich J Gilbert C Ashley B Zhao M Chen H Wu J . Expression of the MHC class II transactivator (CIITA) type IV promoter in b lymphocytes and regulation by IFN-γ. Mol Immunol (2006) 43:519–28. doi: 10.1016/j.molimm.2005.05.005 PMC1482792 15950283

[16] Gao J De BP Banerjee AK . Human parainfluenza virus type 3 up-regulates major histocompatibility complex class I and II expression on respiratory epithelial cells: Involvement of a STAT1- and CIITA-independent pathway. J Virol (1999) 73:1411–8. doi: 10.1128/jvi.73.2.1411-1418.1999 PMC103965 9882346

[17] Hu Z Zhou Q Zhang C Fan S Cheng W Zhao Y . Structural and biochemical basis for induced self-propagation of NLRC4. Science (2015) 350:399–404. doi: 10.1126/science.aac5489 26449475

[18] Zhang L Chen S Ruan J Wu J Tong AB Yin Q . Cryo-EM structure of the activated NAIP2-NLRC4 inflammasome reveals nucleated polymerization. Science (2015) 350:404–9. doi: 10.1126/science.aac5789 PMC4640189 26449474

[19] Kay C Wang R Kirkby M Man SM . Molecular mechanisms activating the NAIP-NLRC4 inflammasome: Implications in infectious disease, autoinflammation, and cancer. Immunol Rev (2020) 297:67–82. doi: 10.1111/imr.12906 32729154

[20] Zhao Y Yang J Shi J Gong YN Lu Q Xu H . The NLRC4 inflammasome receptors for bacterial flagellin and type III secretion apparatus. Nature (2011) 477:596–602. doi: 10.1038/nature10510 21918512

[21] Kupz A Guarda G Gebhardt T Sander LE Short KR Diavatopoulos DA . NLRC4 inflammasomes in dendritic cells regulate noncognate effector function by memory CD8 + T cells. Nat Immunol (2012) 13:162–9. doi: 10.1038/ni.2195 22231517

[22] Freeman L Guo H David CN Brickey WJ Jha S Ting JPY . NLR members NLRC4 and NLRP3 mediate sterile inflammasome activation in microglia and astrocytes. J Exp Med (2017) 214:1351–70. doi: 10.1084/jem.20150237 PMC5413320 28404595

[23] Rauch I Deets K Ji D von Molke J Tenthorey J Lee A . NAIP-NLRC4 inflammasomes coordinate intestinal epithelial cell expulsion with eicosanoid and IL-18 release *via* activation of caspase-1 and -8. Immunity (2017) 46:649–59. doi: 10.1016/j.immuni.2017.03.016 PMC5476318 28410991

[24] Sellin ME Müller AA Felmy B Dolowschiak T Diard M Tardivel A . Epithelium-intrinsic NAIP/NLRC4 inflammasome drives infected enterocyte expulsion to restrict salmonella replication in the intestinal mucosa. Cell Host Microbe (2014) 16:237–48. doi: 10.1016/j.chom.2014.07.001 25121751

[25] Diez E Yaraghi Z MacKenzie A Gros P . The neuronal apoptosis inhibitory protein (Naip) is expressed in macrophages and is modulated after phagocytosis and during intracellular infection with legionella pneumophila. J Immunol (2000) 164:1470–7. doi: 10.4049/jimmunol.164.3.1470 10640764

[26] Bauer R Rauch I . The NAIP/NLRC4 inflammasome in infection and pathology. Mol Aspects Med (2020) 76:100863. doi: 10.1016/j.mam.2020.100863 32499055

[27] Vinzing M Eitel J Lippmann J Hocke AC Zahlten J Slevogt H . NAIP and ipaf control legionella pneumophila replication in human cells. J Immunol (2008) 180:6808–15. doi: 10.4049/jimmunol.180.10.6808 18453601

[28] Inohara N Koseki T Del Peso L Hu Y Yee C Chen S . Nod1, an apaf-1-like activator of caspase-9 and nuclear factor-κB. J Biol Chem (1999) 274:14560–7. doi: 10.1074/jbc.274.21.14560 10329646

[29] Ogura Y Inohara N Benito A Chen FF Yamaoka S Núñez G . Nod2, a Nod1/Apaf-1 family member that is restricted to monocytes and activates NF-κB. J Biol Chem (2001) 276:4812–8. doi: 10.1074/jbc.M008072200 11087742

[30] Gutte PGM Jurt S Grütter MG Zerbe O . Unusual structural features revealed by the solution NMR structure of the NLRC5 caspase recruitment domain. Biochemistry (2014) 53:3106–17. doi: 10.1021/bi500177.x 24815518

[31] Ting JPY Lovering RC Alnemri ESPD Bertin J Boss JM Davis B . The NLR gene family: An official nomenclature. Immunity (2008) 28:285–7. doi: 10.1016/j.immuni.2008.02.005 PMC2630772 18341998

[32] Chin AI Dempsey PW Bruhn K Miller JF Xu Y Cheng G . Involvement of receptor-interacting protein 2 in innate and adaptive immune responses. Nature (2002) 416:190–4. doi: 10.1038/416190a 11894097

[33] Schorey JS Cooper AM . Macrophage signalling upon mycobacterial infection: The MAP kinases lead the way. Cell Microbiol (2003) 5:133–42. doi: 10.1046/j.1462-5822.2003.00263.x 12614457

[34] Franchi L Warner N Viani K Nuñez G . Function of nod-like receptors in microbial recognition and host defense. Immunol Rev (2010) 227:106–28. doi: 10.1111/j.1600-065X.2008.00734.x PMC2679989 19120480

[35] Inohara N Koseki T Lin J Del Peso L Lucas PC Chen FF . An induced proximity model for NF-κB activation in the Nod1/RICK and RIP signaling pathways. J Biol Chem (2000) 275:27823–31. doi: 10.1074/jbc.M003415200 10880512

[36] Hasegawa M Fujimoto Y Lucas PC Nakano H Fukase K Núñez G . A critical role of RICK/RIP2 polyubiquitination in nod-induced NF-κB activation. EMBO J (2008) 27:373–83. doi: 10.1038/sj.emboj.7601962 PMC2234345 18079694

[37] Hsu YMS Zhang Y You Y Wang D Li H Duramad O . The adaptor protein CARD9 is required for innate immune responses to intracellular pathogens. Nat Immunol (2007) 8:198–205. doi: 10.1038/ni1426 17187069

[38] Oviedo-Boyso J Bravo-Patiño A Baizabal-Aguirre VM . Collaborative action of toll-like and nod-like receptors as modulators of the inflammatory response to pathogenic bacteria. Mediators Inflamm (2014) 2014:432785. doi: 10.1155/2014/432785 PMC4267164 25525300

[39] Vegna S Gregoire D Moreau M Lassus P Durantel D Assenat E . NOD1 participates in the innate immune response triggered by hepatitis c virus polymerase. J Virol (2016) 90:6022–35. doi: 10.1128/JVI.03230-15 PMC4907226 27099311

[40] Seth RB Sun L Chen ZJ . Antiviral innate immunity pathways. Cell Res (2006) 16:141–7. doi: 10.1038/sj.cr.7310019 16474426

[41] Sabbah A Chang TH Harnack R Frohlich V Tominaga K Dube P . Activation of innate immune antiviral response by NOD2. Nat Immunol (2009) 110:1073–80. doi: 10.1038/ni.1782 PMC2752345 19701189

[42] Travassos LH Carneiro LAM Ramjeet M Hussey S Kim YG Magalhes JG . Nod1 and Nod2 direct autophagy by recruiting ATG16L1 to the plasma membrane at the site of bacterial entry. Nat Immunol (2010) 11:55–62. doi: 10.1038/ni.1823 19898471

[43] Grubman A Kaparakis M Viala J Allison C Badea L Karrar A . The innate immune molecule, NOD1, regulates direct killing of helicobacter pylori by antimicrobial peptides. Cell Microbiol (2010) 12:626–39. doi: 10.1111/j.1462-5822.2009.01421.x 20039881

[44] Masumoto J Yang K Varambally S Hasegawa M Tomlins SA Qiu S . Nod1 acts as an intracellular receptor to stimulate chemokine production and neutrophil recruitment *in vivo* . J Exp Med (2006) 203:203–13. doi: 10.1084/jem.20051229 PMC2118074 16418393

[45] Girardin SE Jéhanno M Mengin-Lecreulx D Sansonetti PJ Alzari PM Philpott DJ . Identification of the critical residues involved in peptidoglycan detection by Nod1. J Biol Chem (2005) 280:38648–56. doi: 10.1074/jbc.M509537200 16172124

[46] Tanabe T Chamaillard M Ogura Y Zhu L Qiu S Masumoto J . Regulatory regions and critical residues of NOD2 involved in muramyl dipeptide recognition. EMBO J (2004) 23:1587–97. doi: 10.1038/sj.emboj.7600175 PMC391079 15044951

[47] Girardin SE Boneca IG Carneiro LAM Antignac A Jéhanno M Viala J . Nod1 detects a unique muropeptide from gram-negative bacterial peptidoglycan. Science (2003) 300:1584–7. doi: 10.1126/science.1084677 12791997

[48] Chamaillard M Hashimoto M Horie Y Masumoto J Qiu S Saab L . An essential role for NOD1 in host recognition of bacterial peptidoglycan containing diaminopimelic acid. Nat Immunol (2003) 4:702–7. doi: 10.1038/ni945 12796777

[49] Girardin SE Travassos LH Hervé M Blanot D Boneca IG Philpott DJ . Peptidoglycan molecular requirements allowing detection by Nod1 and Nod2. J Biol Chem (2003) 278:41702–8. doi: 10.1074/jbc.M307198200 12871942

[50] Pei G Zyla J He L Moura-Alves P Steinle H Saikali P . Cellular stress promotes NOD1/2-dependent inflammation *via* the endogenous metabolite sphingosine-1-phosphate. EMBO J (2021) 40:1–19. doi: 10.15252/embj.2020106272 PMC8246065 33942347

[51] Keestra-Gounder AM Byndloss MX Seyffert N Young BM Chávez-Arroyo A Tsai AY . NOD1 and NOD2 signalling links ER stress with inflammation. Nature (2016) 532:394–7. doi: 10.1038/nature17631 PMC4869892 27007849

[52] Molinaro R Mukherjee T Flick R Philpott DJ Girardin SE . Trace levels of peptidoglycan in serum underlie the NOD-dependent cytokine response to endoplasmic reticulum stress. J Biol Chem (2019) 294:9007–15. doi: 10.1074/jbc.RA119.007997 PMC6552437 30996003

[53] Karki R Man SM Malireddi RKS Kesavardhana S Zhu Q Burton AR . NLRC3 is an inhibitory sensor of PI3K-mTOR pathways in cancer. Nature (2016) 540(7634):583–7. doi: 10.1038/nature20597 PMC5468516 27951586

[54] Schneider M Zimmermann AG Roberts RA Zhang L Karen V Rahman AH . The innate immune sensor NLRC3 attenuates toll-like receptor signaling *via* modification of the signaling adaptor TRAF6 and transcription factor NF-κB. Nat Immunol (2013) 13:823–31. doi: 10.1038/ni.2378 PMC3721195 22863753

[55] Li X Deng M Petrucelli A Zhu C Mo J Zhang L . Viral DNA binding to NLRC3, an inhibitory nucleic acid sensor, unleashes STING, a cyclic dinucleotide receptor that activates type I interferon. Immunity (2019) 50:591–9. doi: 10.1016/j.immuni.2019.02.009 PMC6469509 30893587

[56] Zhang L Mo J Swanson KV Wen H Petrucelli A Sean M . NLRC3, a member of the NLR family of proteins, is a negative regulator of innate immune signaling induced by the DNA sensor STING. Immunity (2014) 40:329–41. doi: 10.1016/j.immuni.2014.01.010 PMC4011014 24560620

[57] Uchimura T Oyama Y Deng M Guo H Wilson JE Rampanelli E . The innate immune sensor NLRC3 acts as a rheostat that fine-tunes T cell responses in infection and autoimmunity. Immunity (2018) 49:1049–1061.e6. doi: 10.1016/j.immuni.2018.10.008 30566882 PMC6532657

[58] Fu Y Zhan X Wang Y Jiang X Liu M Yang Y . NLRC 3 expression in dendritic cells attenuates CD 4 + T cell response and autoimmunity. EMBO J (2019) 38:1–13. doi: 10.15252/embj.2018101397 PMC6694220 31290162

[59] Gutierrez O Pipaon C Fernandez-Luna JL . Ipaf is upregulated by tumor necrosis factor-α in human leukemia cells. FEBS Lett (2004) 568:79–82. doi: 10.1016/j.febslet.2004.04.095 15196924

[60] Meissner TB Li A Biswas A Lee KH Liu YJ Bayir E . NLR family member NLRC5 is a transcriptional regulator of MHC class I genes. Proc Natl Acad Sci U.S.A. (2010) 107:13794–9. doi: 10.1073/pnas.1008684107 PMC2922274 20639463

[61] Benko S Kovács EG Hezel F Kufer TA . NLRC5 functions beyond MHC I regulation-what do we know so far? Front Immunol (2017) 8:150. doi: 10.3389/fimmu.2017.00150 28261210 PMC5313500

[62] Barton JL Berg T Didon L Nord M . The pattern recognition receptor Nod1 activates CCAAT/enhancer binding protein β signalling in lung epithelial cells. Eur Respir J (2007) 30:214–22. doi: 10.1183/09031936.00143906 17666556

[63] Månsson Kvarnhammar A Tengroth L Adner M Cardell LO . Innate immune receptors in human airway smooth muscle cells: Activation by TLR1/2, TLR3, TLR4, TLR7 and NOD1 agonists. PloS One (2013) 8:1–10. doi: 10.1371/journal.pone.0068701 PMC3701658 23861935

[64] Opitz B Püschel A Schmeck B Hocke AC Rosseau S Hammerschmidt S . Nucleotide-binding oligomerization domain proteins are innate immune receptors for internalized streptococcus pneumoniae. J Biol Chem (2004) 279:36426–32. doi: 10.1074/jbc.M403861200 15215247

[65] Slevogt H Seybold J Tiwari KN Hocke AC Jonatat C Dietel S . Moraxella catarrhalis is internalized in respiratory epithelial cells by a trigger-like mechanism and initiates a TLR2- and partly NOD1-dependent inflammatory immune response. Cell Microbiol (2007) 9:694–707. doi: 10.1111/j.1462-5822.2006.00821.x 17054439

[66] Tada H Aiba S Shibata K . Synergistic effect of Nod1 and Nod2 agonists with toll-like receptor agonists on human dendritic cells to generate interleukin-12 and T helper type 1 cells. Infect (2005) 73:7967–76. doi: 10.1128/IAI.73.12.7967 PMC1307098 16299289

[67] Shimada K Chen S Dempsey PW Sorrentino R Alsabeh R Slepenkin AV . The NOD/RIP2 pathway is essential for host defenses against chlamydophila pneumoniae lung infection. PloS Pathog (2009) 5:e1000379. doi: 10.1371/journal.ppat.1000379 19360122 PMC2660273

[68] Uehara A Fujimoto Y Fukase K Takada H . Various human epithelial cells express functional toll-like receptors, NOD1 and NOD2 to produce anti-microbial peptides, but not proinflammatory cytokines. Mol Immunol (2007) 44:3100–11. doi: 10.1016/j.molimm.2007.02.007 17403538

[69] Yao Y Qian Y . Expression regulation and function of NLRC5. Protein Cell (2013) 4:168–75. doi: 10.1007/s13238-012-2109-3 PMC4875496 23483478

[70] Wang J-Q Liu Y-R Xia Q Chen R-N Liang J Xia Q-R . Emerging roles for NLRC5 in immune diseases. Front Pharmacol (2019) 10:1352. doi: 10.3389/fphar.2019.01352 31824312 PMC6880621

[71] Guo H Callaway JB Ting JPY . Inflammasomes: Mechanism of action, role in disease, and therapeutics. Nat Med (2015) 21:677–87. doi: 10.1038/nm.3893 PMC4519035 26121197

[72] Finger JN Lich JD Dare LC Cook MN Brown KK Duraiswamis C . Autolytic proteolysis within the function to find domain (FIIND) is required for NLRP1 inflammasome activity. J Biol Chem (2012) 287:25030–7. doi: 10.1074/jbc.M112.378323 PMC3408201 22665479

[73] Wang Y Hasegawa M Imamura R Kinoshita T Kondo C Konaka K . PYNOD, a novel apaf-1/CED4-like protein is an inhibitor of ASC and caspase-1. Int Immunol (2004) 16:777–86. doi: 10.1093/intimm/dxh081 15096476

[74] Martinon F Burns K Tschopp J . The inflammasome: A molecular platform triggering activation of inflammatory caspases and processing of proIL-β. Mol Cell (2002) 10:417–26. doi: 10.1016/S1097-2765(02)00599-3 12191486

[75] Sandstron A Mitchell P Goers L Mu E Lesser C Vance R . Functional degradation: a mechanism of NLRP1 inflammasome activation by diverse pathogen enzymes. Science (2019) 364:6435.eaau1330. doi: 10.1126/science.aau1330 PMC6532986 30872533

[76] Mitchell P Sandstrom A Vance R . The NLRP1 inflammasome: new mechanistic insights and unresolved mysteries. Curr Opin Immunol (2019) 60:37–45. doi: 10.1016/j.coi.2019.04.015 31121538 PMC6800612

[77] Swanson KV Deng M Ting JPY . The NLRP3 inflammasome: molecular activation and regulation to therapeutics. Nat Rev Immunol (2019) 19:477–89. doi: 10.1038/s41577-019-0165-0 PMC7807242 31036962

[78] Yang Y Wang H Kouadir M Song H Shi F . Recent advances in the mechanisms of NLRP3 inflammasome activation and its inhibitors. Cell Death Dis (2019) 10(2):128. doi: 10.1038/s41419-019-1413-8 PMC6372664 30755589

[79] Bauernfeind F Horvath G Stutz A Alnemri ES Speert D Fernandes-alnemri T . NF-kB activating pattern recognition and cytokine receptors license NLRP3 inflammasome activation by regulating NLRP3 expression. J Immunol (2009) 183:787–91. doi: 10.4049/jimmunol.0901363 PMC2824855 19570822

[80] Duncan JA Bergstralh DT Wang Y Willingham SB Ye Z Zimmermann AG . Cryopyrin/NALP3 binds ATP/dATP, is an ATPase, and requires ATP binding to mediate inflammatory signaling. Proc Natl Acad Sci U.S.A. (2007) 104:8041–6. doi: 10.1073/pnas.0611496104 PMC1876568 17483456

[81] Lu A Magupalli V Ruan J Yin Q Maninjay K Vos M . Unified polymerization mechanism for the assembly of ASC-dependent inflammasomes. Cell (2014) 156:1193–206. doi: 10.1016/j.cell.2014.02.008 PMC4000066 24630722

[82] Boucher D Monteleone M Coll RC Chen KW Ross CM Teo JL . Caspase-1 self-cleavage is an intrinsic mechanism to terminate inflammasome activity. J Exp Med (2018) 215:827–40. doi: 10.1084/jem.20172222 PMC5839769 29432122

[83] Fernandes-Alnemri T Wu J Yu JW Datta P Miller B Jankowski W . The pyroptosome: A supramolecular assembly of ASC dimers mediating inflammatory cell death *via* caspase-1 activation. Cell Death Differ (2007) 14:1590–604. doi: 10.1038/sj.cdd.4402194 PMC3345951 17599095

[84] Chu ZL Pio F Xie Z Welsh K Krajewska M Krajewski S . A novel enhancer of the Apaf1 apoptosome involved in cytochrome c-dependent caspase activation and apoptosis. J Biol Chem (2001) 276:9239–45. doi: 10.1074/jbc.M006309200 11113115

[85] Kummer JA Broekhuizen R Everett H Agostini L Kuijk L Martinon F . Inflammasome components NALP 1 and 3 show distinct but separate expression profiles in human tissues suggesting a site-specific role in the inflammatory response. J Histochem Cytochem (2007) 55:443–52. doi: 10.1369/jhc.6A7101.2006 17164409

[86] Agostini L Martinon F Burns K Mcdermott MF Hawkins PN Rg Tschopp J . NALP3 forms an IL-1-Processing inflammasome with increased activity in muckle-wells autoinflammatory disorder containing protein called ASC binds and activates pro-caspase-1 (Martinon ASC contains a c-terminal CARD motif as well as an n-terminal CAR. Immunity (2004) 20:319–25. doi: 10.1016/S1074-7613(04)00046-9 15030775

[87] Arnoult D Soaras F Tattoli I Castanier C Philpott DJ Girardin SE . An n-terminal addressing sequence targets NLRX1 to the mitochondrial matrix. J Cell Sci (2009) 122:3161–8. doi: 10.1242/JCS.051193 PMC2871076 19692591

[88] Reubold TF Hahne G Wohlgemuth S Eschenburg S . Crystal structure of the leucine-rich repeat domain of the NOD-like receptor NLRP1: Implications for binding of muramyl dipeptide. FEBS Lett (2014) 588:3327–32. doi: 10.1016/j.febslet.2014.07.017 25064844

[89] Nagai-Singer MA Morrison HA Allen IC . NLRX1 is a multifaceted and enigmatic regulator of immune system function. Front Immunol (2019) 10:2419. doi: 10.3389/fimmu.2019.02419 31681307 PMC6797603

[90] Moore CB Bergstralh DT Duncan JA Lei Y Morrison TE Zimmermann AG . NLRX1 is a regulator of mitochondrial antiviral immunity. Nature (2008) 451:573–7. doi: 10.1038/nature06501 18200010

[91] Bae JS Pasaje CFA Park BL Cheong HS Kim JH Uh ST . Genetic association analysis of CIITA variations with nasal polyp pathogenesis in asthmatic patients. Mol Med Rep (2013) 7:927–34. doi: 10.3892/mmr.2012.1251 23292525

[92] Hysi P Kabesch M Moffatt MF Schedel M Carr D Zhang Y . NOD1 variation, immunoglobulin e and asthma. Hum Mol Genet (2005) 14:935–41. doi: 10.1093/hmg/ddi087 15718249

[93] Weidinger S Klopp N Rümmler L Wagenpfeil S Baurecht HJ Gauger A . Association of CARD15 polymorphisms with atopy-related traits in a population-based cohort of Caucasian adults. Clin Exp Allergy (2005) 35:866–72. doi: 10.1111/j.1365-2222.2005.02269.x 16008671

[94] Rossios C Pavlidis S Hoda U Kuo CH Wiegman C Russell K . Sputum transcriptomics reveal upregulation of IL-1 receptor family members in patients with severe asthma. J Allergy Clin Immunol (2018) 141:560–70. doi: 10.1016/j.jaci.2017.02.045 28528200

[95] Leal VNC Genov IR Mallozi MC Solé D Pontillo A . Polymorphisms in inflammasome genes and risk of asthma in Brazilian children. Mol Immunol (2018) 93:64–7. doi: 10.1016/j.molimm.2017.11.006 29154202

[96] Hitomi Y Ebisawa M Tomikawa M Imai T Komata T Hirota T . Associations of functional NLRP3 polymorphisms with susceptibility to food-induced anaphylaxis and aspirin-induced asthma. J Allergy Clin Immunol (2009) 124:779–785.e6. doi: 10.1016/j.jaci.2009.07.044 19767079

[97] Steimle V Otten LA Zufferey M Mach B . Complementation cloning of an MHC class II transactivator mutated in hereditary MHC class II deficiency (or bare lymphocyte syndrome). Cell (1993) 75:135–46. doi: 10.1016/S0092-8674(05)80090-X 8402893

[98] Saleem MA Arkwright PD Davies EG Cant AJ Veys PA . Clinical course of patients with major histocompatibility complex class II deficiency. Arch Dis Child (2000) 83:356–9. doi: 10.1136/adc.83.4.356 PMC1718526 10999878

[99] Lum SH Neven B Slatter MA Gennery AR . Hematopoietic cell transplantation for MHC class II deficiency. Front Pediatr (2019) 7:516. doi: 10.3389/fped.2019.00516 31921728 PMC6917634

[100] Chang CH Guerder S Hong SC Van Ewijk W Flavell RA . Mice lacking the MHC class II transactivator (CIITA) show tissue-specific impairment of MHC class II expression. Immunity (1996) 4:167–78. doi: 10.1016/S1074-7613(00)80681-0 8624807

[101] Demenais F Margaritte-Jeannin P Barnes K Al. E . Multiancestry association study identifies new asthma risk loci that colocalize with immune cell enhancer marks. Nat Genet (2018) 50:42–53. doi: 10.1038/s41588-017-0014-7 29273806 PMC5901974

[102] Ferreira MAR Mathur R Vonk JM Szwajda A Brumpton B Granell R . Genetic architectures of childhood- and adult-onset asthma are partly distinct. Am J Hum Genet (2019) 104:665–84. doi: 10.1016/j.ajhg.2019.02.022 PMC6451732 30929738

[103] Eder W Klimecki W Yu L Von Mutius E Riedler J Braun-Fahrländer C . Association between exposure to farming, allergies and genetic variation in CARD4/NOD1. Allergy Eur J Allergy Clin Immunol (2006) 61:1117–24. doi: 10.1111/j.1398-9995.2006.01128.x 16918516

[104] Belhaj R Kaabachi W Khalfallah I Hamdi B Hamzaoui K Hamzaoui A . Gene variants, mRNA and NOD1/2 protein levels in Tunisian childhood asthma. Lung (2019) 197:377–85. doi: 10.1007/s00408-019-00209-4 30874883

[105] Kabesch M Peters W Carr D Leupold W Weiland SK Von Mutius E . Association between polymorphisms in caspase recruitment domain containing protein 15 and allergy in two German populations. J Allergy Clin Immunol (2003) 111:813–7. doi: 10.1067/mai.2003.1336 12704363

[106] Weidinger S Klopp N Rummler L Wagenpfeil S Novak N Baurecht HJ . Association of NOD1 polymorphisms with atopic eczema and related phenotypes. J Allergy Clin Immunol (2005) 116:177–84. doi: 10.1016/j.jaci.2005.02.034 15990792

[107] Cai X Xu Q Zhou C Zhou L Dai W Ji G . The association of nucleotide-binding oligomerization domain 2 gene polymorphisms with the risk of asthma in the Chinese han population. Mol Genet Genomic Med (2019) 7:1–7. doi: 10.1002/mgg3.675 PMC6565575 30950247

[108] Moecking J Laohamonthonkul P Chalker K White MJ Harapas CR Yu CH . NLRP1 variant M1184V decreases inflammasome activation in the context of DPP9 inhibition and asthma severity. J Allergy Clin Immunol (2021) 147:2134–2145.e20. doi: 10.1016/j.jaci.2020.12.636 33378691 PMC8168955

[109] Queiroz G de A da Silva RR Pires A de O Costa R dos S Alcântara-Neves NM da Silva TM . New variants in NLRP3 inflammasome genes increase risk for asthma and blomia tropicalis-induced allergy in a Brazilian population. Cytokine X (2020) 2:100032. doi: 10.1016/j.cytox.2020.100032 33015616 PMC7522708

[110] Laitinen T Daly MJ Rioux JD Kauppi P Laprise C Petäys T . A susceptibility locus for asthma-related traits on chromosome 7 revealed by genome-wide scan in a founder population. Nat Genet (2001) 28:87–91. doi: 10.1038/88319 11326283

[111] Daniels SE Bhattacharrya S James A Leaves NI Young A Hill MR . A genome-wide search for quantitative trait loci underlying asthma. Nature (1996) 383:247–50. doi: 10.1038/383247a0 8805698

[112] Farkas L Stoelcker B Jentsch N Heitzer S Pfeifer M Schulz C . Muramyldipeptide modulates CXCL-8 release of BEAS-2B cells *via* NOD2. Scand J Immunol (2008) 68:315–22. doi: 10.1111/j.1365-3083.2008.02145.x 18647246

[113] Wong CK Hu S Leung KM-L Dong J He L Chu YJ . NOD-like receptors mediated activation of eosinophils interacting with bronchial epithelial cells: A link between innate immunity and allergic asthma. Cell Mol Immunol (2013) 10:317–29. doi: 10.1038/cmi.2012.77 PMC4003204 23524653

[114] Ait Yahia S Audousset C Alvarez-Simon D Vorng H Togbe D Marquillies P . NOD1 sensing of house dust mite–derived microbiota promotes allergic experimental asthma. J Allergy Clin Immunol (2021) 148:394–406. doi: 10.1016/j.jaci.2020.12.649 33508265

[115] Bauer RN Brighton LE Mueller L Xiang Z Rager JE Fry RC . Influenza enhances caspase-1 in bronchial epithelial cells from asthmatic volunteers and is associated with pathogenesis. J Allergy Clin Immunol (2012) 130(4):958–67.e14. doi: 10.1016/j.jaci.2012.07.013 23021143 PMC3470476

[116] Zuyderduyn S Sukkar MB Fust A Dhaliwal S Burgess JK . Treating asthma means treating airway smooth muscle cells. Eur Respir J (2008) 32:265–74. doi: 10.1183/09031936.00051407 18669785

[117] Ni G Chen Y Wu F Zhu P Song L . NOD2 promotes cell proliferation and inflammatory response by mediating expression of TSLP in human airway smooth muscle cells. Cell Immunol (2017) 312:35–41. doi: 10.1016/j.cellimm.2016.11.007 27889082

[118] Tourneur E Ben Mkaddem S Chassin C Bens M Goujon JM Charles N . Cyclosporine a impairs nucleotide binding oligomerization domain (Nod1)-mediated innate antibacterial renal defenses in mice and human transplant recipients. PloS Pathog (2013) 9:e1003152. doi: 10.1371/journal.ppat.1003152 23382681 PMC3561241

[119] Gutierrez O Pipaon C Inohara N Fontalba A Ogura Y Prosper F . Induction of Nod2 in myelomonocytic and intestinal epithelial cells *via* nuclear factor-κB activation. J Biol Chem (2002) 277:41701–5. doi: 10.1074/jbc.M206473200 12194982

[120] Kvarnhammar AM Petterson T Cardell LO . NOD-like receptors and RIG-i-like receptors in human eosinophils: Activation by NOD1 and NOD2 agonists. Immunology (2011) 134:314–25. doi: 10.1111/j.1365-2567.2011.03492.x PMC3209571 21978001

[121] Wong CK Leung TF Chu IMT Dong J Lam YYO Lam CWK . Aberrant expression of regulatory cytokine IL-35 and pattern recognition receptor NOD2 in patients with allergic asthma. Inflammation (2014) 38:348–60. doi: 10.1007/s10753-014-0038-4 25326182

[122] Ekman AK Cardell LO . The expression and function of nod-like receptors in neutrophils. Immunology (2010) 130:55–63. doi: 10.1111/j.1365-2567.2009.03212.x 20002790 PMC2855793

[123] Clarke TB Davis KM Lysenko ES Zhou AY Yu Y Weiser JN . Recognition of peptidoglycan from the microbiota by Nod1 enhances systemic innate immunity. Nat Med (2010) 16:228–31. doi: 10.1038/nm.2087 PMC4497535 20081863

[124] Fritz JH Girardin SE Fitting C Werts C Mengin-Lecreulx D Caroff M . Synergistic stimulation of human monocytes and dendritic cells by toll-like receptor 4 and NOD1- and NOD2-activating agonists. Eur J Immunol (2005) 35:2459–70. doi: 10.1002/eji.200526286 16021602

[125] Ait Yahia S Azzaoui I Everaere L Vorng H Chenivesse C Marquillies P . CCL17 production by dendritic cells is required for NOD1-mediated exacerbation of allergic asthma. Am J Respir Crit Care Med (2014) 189:899–908. doi: 10.1164/rccm.201310-1827OC 24661094

[126] Fritz JH Le Bourhis L Sellge G Magalhaes JG Fsihi H Kufer TA . Nod1-mediated innate immune recognition of peptidoglycan contributes to the onset of adaptive immunity. Immunity (2007) 26:445–59. doi: 10.1016/j.immuni.2007.03.009 17433730

[127] Magalhaes JG Fritz JH Le Bourhis L Sellge G Travassos LH Selvanantham T . Nod2-dependent Th2 polarization of antigen-specific immunity. J Immunol (2008) 181:7925–35. doi: 10.4049/jimmunol.181.11.7925 19017983

[128] Magalhaes JG Rubino SJ Travassos LH Le Bourhis L Duan W Sellge G . Nucleotide oligomerization domain-containing proteins instruct T cell helper type 2 immunity through stromal activation. Proc Natl Acad Sci U.S.A. (2012) 109:10605. doi: 10.1073/pnas.1208781109 PMC3169112 21856952

[129] Shaw MH Reimer T Sánchez-Valdepeñas C Warner N Kim Y-G Fresno M . T Cell intrinsic role of Nod2 in promoting type I immunity against toxoplasma gondii. Nat Immunol (2009) 10(12):1267–74. doi: 10.1038/ni.1816 PMC2803073 19881508

[130] Napier RJ Lee EJ Davey MP Vance EE Furtado JM Snow PE . T Cell-intrinsic role for Nod2 in protection against Th17-mediated uveitis. Nat Commun (2020) 11(1):1–16. doi: 10.1038/s41467-020-18961-0 33106495 PMC7589501

[131] Nakashima K Hirota T Suzuki Y Akahoshi M Shimizu M Jodo A . Association of the RIP2 gene with childhood atopic asthma. Allergol. Int (2006) 55:77–83. doi: 10.2332/allergolint.55.77 17075290

[132] Goh FY Cook KLTP Upton N Tao L Lah LC Leung BP . Receptor-interacting protein 2 gene silencing attenuates allergic airway inflammation. J Immunol (2013) 191:2691–9. doi: 10.4049/jimmunol.1202416 23918989

[133] Kim TH Park YM Ryu SW Kim DJ Park JH Park JH . Receptor interacting protein 2 (RIP2) is dispensable for OVA-induced airway inflammation in mice. Allergy Asthma Immunol Res (2014) 6:163–8. doi: 10.4168/aair.2014.6.2.163 PMC3936046 24587954

[134] Miller MH Shehat MG Alcedo KP Spinel LP Soulakova J Tigno-Aranjuez JT . Frontline science: RIP2 promotes house dust mite-induced allergic airway inflammation. J Leukoc Biol (2018) 140(3):1–13. doi: 10.1002/JLB.4HI0118-017RR PMC6113092 30052281

[135] Tan HTT Hagner S Ruchti F Radzikowska U Tan G Altunbulakli C . Tight junction, mucin, and inflammasome-related molecules are differentially expressed in eosinophilic, mixed, and neutrophilic experimental asthma in mice. Allergy Eur J Allergy Clin Immunol (2019) 74:294–307. doi: 10.1111/all.13619 30267575

[136] Birrell MA Eltom S . The role of the NLRP3 inflammasome in the pathogenesis of airway disease. Pharmacol Ther (2011) 130:364–70. doi: 10.1016/j.pharmthera.2011.03.007 21421008

[137] Hirota JA Hirota SA Warner SM Stefanowicz D Shaheen F Beck PL . The airway epithelium nucleotide-binding domain and leucine-rich repeat protein 3 inflammasome is activated by urban particulate matter. J Allergy Clin Immunol (2012) 129:1116–1125.e6. doi: 10.1016/j.jaci.2011.11.033 22227418

[138] Tsai YM Chiang KH Hung JY Chang WA Lin HP Shieh JM . Der f1 induces pyroptosis in human bronchial epithelia *via* the NLRP3 inflammasome. Int J Mol Med (2018) 41:757–64. doi: 10.3892/ijmm.2017.3310 PMC5752164 29207030

[139] Kim SR Kim DI Kim SH Lee H Lee KS Cho SH . NLRP3 inflammasome activation by mitochondrial ROS in bronchial epithelial cells is required for allergic inflammation. Cell Death Dis (2014) 5:1–15. doi: 10.1038/cddis.2014.460 PMC4237270 25356867

[140] Kim RY Pinkerton JW Essilfie AT Robertson AAB Baines KJ Brown AC . Role for NLRP3 inflammasome-mediated, IL-1β-dependent responses in severe, steroid-resistant asthma. Am J Respir Crit Care Med (2017) 196:283–97. doi: 10.1164/rccm.201609-1830OC 28252317

[141] Simpson JL Phipps S Baines KJ Oreo KM Gunawardhana L Gibson PG . Elevated expression of the NLRP3 inflammasome in neutrophilic asthma. Eur Respir J (2014) 43:1067–76. doi: 10.1183/09031936.00105013 24136334

[142] Gordon EM Yao X Xu H Karkowsky W Kaler M Kalchiem-Dekel O . Apolipoprotein e is a concentration-dependent pulmonary danger signal that activates the NLRP3 inflammasome and IL-1β secretion by bronchoalveolar fluid macrophages from asthmatic subjects. J Allergy Clin Immunol (2019) 144:426–441.e3. doi: 10.1016/j.jaci.2019.02.027 30872118 PMC9152878

[143] Doitsh G Galloway NLKK Geng X Yang Z Monroe KM Zepeda O . Pyroptosis drives CD4 T-cell depletion. Nature (2014) 505:509–14. doi: 10.1038/nature12940 PMC4047036 24356306

[144] Bruchard M Rebé C Derangère V Togbé D Ryffel B Boidot R . The receptor NLRP3 is a transcriptional regulator of TH2 differentiation. Nat Immunol (2015) 16:859–70. doi: 10.1038/ni.3202 26098997

[145] Kool M Willart MAM van Nimwegen M Bergen I Pouliot P Virchow JC . An unexpected role for uric acid as an inducer of T helper 2 cell immunity to inhaled antigens and inflammatory mediator of allergic asthma. Immunity (2011) 34:527–40. doi: 10.1016/j.immuni.2011.03.015 21474346

[146] Allen IC Jania CM Wilson JE Tekeppe EM Hua X Brickey WJ . Analysis of NLRP3 in the development of allergic airway disease in mice. J Immunol (2012) 188:2884–93. doi: 10.4049/jimmunol.1102488 PMC3294123 22323538

[147] Madouri F Guillou N Fauconnier L Marchiol T Rouxel N Madouri F . Caspase-1 activation by NLRP3 inflammasome dampens IL-33-dependent house dust mite-induced allergic lung inflammation. J Mol Cell Biol (2015) 7:351–65. doi: 10.1093/jmcb/mjv012 25714839

[148] Eisenbarth SC Colegio OR O’Connor W Sutterwala FS Flavell RA . Crucial role for the Nalp3 inflammasome in the immunostimulatory properties of aluminium adjuvants. Nature (2008) 453:1122–6. doi: 10.1038/nature06939 PMC4804622 18496530

[149] Besnard AG Guillou N Tschopp J Erard F Couillin I Iwakura Y . NLRP3 inflammasome is required in murine asthma in the absence of aluminum adjuvant. Allergy Eur J Allergy Clin Immunol (2011) 66:1047–57. doi: 10.1111/j.1398-9995.2011.02586.x 21443539

[150] Kim HY Lee HJ Chang YJ Pichavant M Shore SA Fitzgerald KA . Interleukin-17-producing innate lymphoid cells and the NLRP3 inflammasome facilitate obesity-associated airway hyperreactivity. Nat Med (2014) 20(1):54–61. doi: 10.1038/nm.3423 24336249 PMC3912313

[151] Besnard A Togbe D Couillin I Tan Z Zheng SG Erard F . Inflammasome – IL- 1 – Th 17 response in allergic lung inflammation. J Mol Cell Biol (2012) 4:3–10. doi: 10.1093/jmcb/mjr042 22147847

[152] Sebag SC Koval OM Paschke JD Winters CJ Jaffer OA Dworski R . Mitochondrial CaMKII inhibition in airway epithelium protects against allergic asthma. JCI Insight (2017) 2:1–16. doi: 10.1172/jci.insight.88297 PMC5291733 28194433

[153] Miller MH Shehat MG Tigno-Aranjuez JT . Immune modulation of allergic asthma by early pharmacological inhibition of RIP2. ImmunoHorizons (2020) 4:825–36. doi: 10.4049/immunohorizons.2000073 PMC7895314 33443037

[154] Primiano MJ Lefker BA Bowman MR Bree AG Hubeau C Bonin PD . Efficacy and pharmacology of the NLRP3 inflammasome inhibitor CP-456,773 (CRID3) in murine models of dermal and pulmonary inflammation. J Immunol (2016) 197:2421–33. doi: 10.4049/jimmunol.1600035 27521339

[155] Wang L Zha B Shen Q Zou H Cheng C Wu H . Sevoflurane inhibits the Th2 response and NLRP3 expression in murine allergic airway inflammation. J Immunol Res (2018) 2018. doi: 10.1155/2018/9021037 PMC6186358 30363922

[156] Lv J Su W Yu Q Zhang M Di C Lin X . Heme oxygenase-1 protects airway epithelium against apoptosis by targeting the proinflammatory NLRP3–RXR axis in asthma. J Biol Chem (2018) 293:18454–65. doi: 10.1074/jbc.RA118.004950 PMC6290169 30333233

[157] Chen S Yao L Huang P He Q Guan H Luo Y . Blockade of the NLRP3/Caspase-1 axis ameliorates airway neutrophilic inflammation in a toluene diisocyanate-induced murine asthma model. Toxicol Sci (2019) 170:462–75. doi: 10.1093/toxsci/kfz099 31070765

[158] Lunding LP Skouras DB Vock C Dinarello CA Wegmann M . The NLRP3 inflammasome inhibitor, OLT1177 ® , ameliorates experimental allergic asthma in mice. Allergy (2021) 77(3):1–4. doi: 10.1111/all.15164 34716997

[159] Qin W Wu X Jia Y Tong X Guo C Chen D . Suhuang antitussive capsule inhibits NLRP3 inflammasome activation and ameliorates pulmonary dysfunction *via* suppression of endoplasmic reticulum stress in cough variant asthma. BioMed Pharmacother (2019) 118:109188. doi: 10.1016/j.biopha.2019.109188 31315072

[160] Liu X Shen J Fan D Qiu X Guo Q Zheng K . Yupingfeng San inhibits NLRP3 inflammasome to attenuate the inflammatory response in asthma mice. Front Pharmacol (2017) 8:944. doi: 10.3389/fphar.2017.00944 29311942 PMC5743824

[161] Guan M Ma H Fan X Chen X Miao M Wu H . Dexamethasone alleviate allergic airway inflammation in mice by inhibiting the activation of NLRP3 inflammasome. Int Immunopharmacol (2020) 78:106017. doi: 10.1016/j.intimp.2019.106017 31780368

[162] Hugot J Chamaillard M Zouali H Lesage S Ce J Macry J . Association of NOD2 leucine-rich repeat variants with susceptibility to crohn’s disease. Nature (2001) 411:599–603. doi: 10.1038/35079107 11385576

[163] Tigno-Aranjuez JT Benderitter P Rombouts F Deroose F Bai XD Mattioli B . *In vivo* inhibition of RIPK2 kinase alleviates inflammatory disease. J Biol Chem (2014) 289:29651–64. doi: 10.1074/jbc.M114.591388 PMC4207980 25213858

[164] Haile PA Votta BJ Marquis RW Bury MJ Mehlmann JF Singhaus R . The identification and pharmacological characterization of 6-(tert-Butylsulfonyl)-N-(5-fluoro-1H-indazol-3-yl)quinolin-4-amine (GSK583), a highly potent and selective inhibitor of RIP2 kinase. J Med Chem (2016) 59:4867–80. doi: 10.1021/acs.jmedchem.6b00211 27109867

[165] Nachbur U Stafford CA Bankovacki A Zhan Y Lindqvist LM Fiil BK . A RIPK2 inhibitor delays NOD signalling events yet prevents inflammatory cytokine production. Nat Commun (2015) 6. doi: 10.1038/ncomms7442 25778803

[166] Goncharov T Hedayati S Mulvihill MM Izrael-Tomasevic A Zobel K Jeet S . Disruption of XIAP-RIP2 association blocks NOD2-mediated inflammatory signaling. Mol Cell (2018) 69:648–663.e7. doi: 10.1016/j.molcel.2018.01.016 29452636

[167] Haile PA Casillas LN Bury MJ Mehlmann JF Singhaus R Charnley AK . Identification of quinoline-based RIP2 kinase inhibitors with an improved therapeutic index to the hERG ion channel. ACS Med Chem Lett (2018) 9:1039–44. doi: 10.1021/acsmedchemlett.8b00344 PMC6187414 30344914

[168] Perregaux DG Mcniff P Laliberte R Hawryluk N Peurano H Stam E . Identification and characterization of a novel class of interleukin-1 post-translational processing inhibitors. J Pharmacol Exp Ther (2001) 299:187–97. 11561079

[169] Coll RC Hill JR Day CJ Zamoshnikova A Boucher D Massey NL . MCC950 directly targets the NLRP3 ATP-hydrolysis motif for inflammasome inhibition. Nat Chem Biol (2019) 15:556–9. doi: 10.1038/s41589-019-0277-7 31086327

[170] Tapia-abellán A Angosto-bazarra D Martínez-banaclocha H De C . MCC950 closes the active conformation of NLRP3 to an inactive state. Nat Chem Biol (2019) 15:560–4. doi: 10.1038/s41589-019-0278-6.MCC950 PMC7116292 31086329

[171] Marchetti C Swartzwelter B Gamboni F Neff CP Richter K Azam T . OLT1177, a β-sulfonyl nitrile compound, safe in humans, inhibits the NLRP3 inflammasome and reverses the metabolic cost of inflammation. Proc Natl Acad Sci U.S.A. (2018) 115:E1530–9. doi: 10.1073/pnas.1716095115 PMC5816172 29378952

[172] Tomani JCD Kagisha V Tchinda AT Jansen O Ledoux A Vanhamme L . The inhibition of NLRP3 inflammasome and IL-6 production by hibiscus noldeae baker f. derived constituents provides a link to its anti-inflammatory therapeutic potentials. Molecules (2020) 25:1–16. doi: 10.3390/molecules25204693 PMC7587372 33066442

[173] Ritter M Straubinger K Schmidt S Busch DH Hagner S Garn H . Functional relevance of NLRP3 inflammasome-mediated interleukin (IL)-1β during acute allergic airway inflammation. Clin Exp Immunol (2014) 178:212–23. doi: 10.1111/cei.12400 PMC4233370 24943899

[174] Hernandez ML Mills K Almond M Todoric K Aleman MM Zhang H . IL-1 receptor antagonist reduces endotoxin-induced airway inflammation in healthy volunteers. J Allergy Clin Immunol (2015) 135:379–85. doi: 10.1016/j.jaci.2014.07.039 PMC4323893 25195169

[175] Kapur S Bonk ME Caspi A . Rilonacept (arcalyst), an interleukin-I trap for the treatment of cryopyrin-associated periodic syndromes. Pharm Ther (2009) 34:138–41. PMC2697082 19561849

[176] Lachmann HJ Kone-Paut I Kuemmerle-Deschner JB Leslie KS Hachulla E Quartier P . Use of canakinumab in the cryopyrin-associated periodic syndrome. N Engl J Med (2009) 360:2416–25. doi: 10.1056/nejmoa0810787 19494217

